# Conservation and Evolution of *Cis*-Regulatory Systems in Ascomycete Fungi

**DOI:** 10.1371/journal.pbio.0020398

**Published:** 2004-11-09

**Authors:** Audrey P Gasch, Alan M Moses, Derek Y Chiang, Hunter B Fraser, Mark Berardini, Michael B Eisen

**Affiliations:** **1**Genome Sciences Department, Genomics Division, Lawrence Berkeley National Laboratory, Berkeley, California, United States of America; **2**Graduate Group in Biophysics, University of CaliforniaBerkeley, CaliforniaUnited States of America; **3**Department of Molecular and Cell Biology, University of CaliforniaBerkeley, CaliforniaUnited States of America; **4**Life Science Division, Lawrence Berkeley National LaboratoryBerkeley, CaliforniaUnited States of America

## Abstract

Relatively little is known about the mechanisms through which gene expression regulation evolves. To investigate this, we systematically explored the conservation of regulatory networks in fungi by examining the *cis*-regulatory elements that govern the expression of coregulated genes. We first identified groups of coregulated Saccharomyces cerevisiae genes enriched for genes with known upstream or downstream *cis*-regulatory sequences. Reasoning that many of these gene groups are coregulated in related species as well, we performed similar analyses on orthologs of coregulated S. cerevisiae genes in 13 other ascomycete species. We find that many species-specific gene groups are enriched for the same flanking regulatory sequences as those found in the orthologous gene groups from S. cerevisiae, indicating that those regulatory systems have been conserved in multiple ascomycete species. In addition to these clear cases of regulatory conservation, we find examples of *cis*-element evolution that suggest multiple modes of regulatory diversification, including alterations in transcription factor-binding specificity, incorporation of new gene targets into an existing regulatory system, and cooption of regulatory systems to control a different set of genes. We investigated one example in greater detail by measuring the in vitro activity of the S. cerevisiae transcription factor Rpn4p and its orthologs from Candida albicans and Neurospora crassa. Our results suggest that the DNA binding specificity of these proteins has coevolved with the sequences found upstream of the Rpn4p target genes and suggest that Rpn4p has a different function in N. crassa.

## Abstract 2

A systematic examination of the gene regulatory elements in ascomycete fungi reveals striking conservation along with some examples of the ways in which regulatory systems can evolve

## Introduction

The diversity of modern organisms reflects and arises from an underlying molecular diversity that is only beginning to be understood. In recent years, much focus has been given to the evolution of protein coding regions, under the assumption that diversification of protein function has driven the evolution of organismal form and function. Nevertheless, the relative dearth of species-specific genes, and the seeming abundance of functionally homologous proteins in many different genomes, suggest additional mechanisms of diversification. One mechanism likely to play a significant role is variation in gene expression (Monod and Jacob 1961; Wilson et al. 1974). Subtle alterations in the timing, location, and levels of protein synthesis can have considerable consequences at both the molecular and organismal level (Averof and Patel 1997; Gompel and Carroll 2003; Lee et al. 2003). Despite the likely importance of variation in gene expression, relatively little is known about the evolution of gene-expression regulation or how this evolution contributes to organismal diversification.

Much of a gene's expression pattern is dictated by flanking noncoding sequences that contain, among other things, binding sites recognized by sequence-specific nucleotide-binding proteins that modulate transcript abundance. A number of recent studies have examined the evolution of *cis*-regulatory elements in alignments of orthologous regulatory regions, consistently showing that these elements evolve at a slower rate than the nonfunctional DNA that surrounds them (Hardison et al. 1997; Loots et al. 2000; McGuire et al. 2000; Bergman and Kreitman 2001; Dermitzakis and Clark 2002; Rajewsky et al. 2002; Moses et al. 2003). Most of these studies have been limited to closely related species whose orthologous noncoding sequences can be aligned, such that the putative*cis*-regulatory elements can be identified and compared. *Cis*-regulatory elements can be conserved in more distantly related species, even when the orthologous regulatory regions are too divergent to be accurately aligned (Piano et al. 1999; Cliften et al. 2003; Romano and Wray 2003). However, without the guidance of multiple alignments, little has been gleaned about the patterns of evolution or the functional constraints that act on *cis*-regulatory elements over longer evolutionary timescales.

Recently, several methods have been developed to dissect the regulatory networks that function within an individual species. Myriad studies have shown that functional regulatory sequences can be identified in a set of coregulated genes on the basis of the enriched fraction of those genes that contain the sequence within their flanking regions (van Helden et al. 1998; Tavazoie et al. 1999; McGuire et al. 2000; Bussemaker et al. 2001; Sinha and Tompa 2002). Gene coregulation can be conserved in related species, and this conservation has been exploited for the computational prediction of *cis*-regulatory elements that are highly conserved (Gelfand et al. 2000; Qin et al. 2003; Wang and Stormo, 2003; Pritsker et al. 2004; Yu et al. 2004). We reasoned that we could extend this approach to examine the evolution of *cis*-regulatory networks across species, by analyzing the orthologs of genes coregulated in S. cerevisiae.

**Figure 1:**
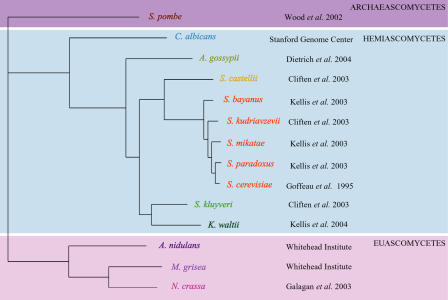
Fungal Phylogeny The phylogenetic tree shows the 14 different fungi analyzed in this study. The topology of the tree was based on Kurtzman and Robnett (2003), and the branch lengths represent the average of maximum-likelihood estimates of synonymous amino acid substitutions (obtained using the PAML package [Yang 1997]) for the 303 proteins that had orthologs assigned in all 14 of these genomes. The closely related saccharomycete species for which the orthologous upstream regions can be aligned are labeled in orange. The source of each genome sequence is also indicated to the right of each species.

As a first step toward this goal, we have examined the simplest model of regulatory networks: the connection between groups of coregulated genes and the flanking *cis*-regulatory sequences that coordinate their expression. We characterized groups of coregulated S. cerevisiae genes and their orthologs in 13 additional ascomycete fungi (Figure 1) and assessed the enriched fraction of those genes that contain known and novel *cis*-regulatory sequences. Our results strongly suggest that many of the known*cis*-regulatory systems from S. cerevisiae have been conserved over hundreds of millions of years of evolution (Berbee and Taylor 1993; Heckman et al. 2001). Based on these observations, we present a number of models for the mechanisms of *cis*-regulatory evolution.

## Results

We began by systematically characterizing known *cis*-regulatory elements and their gene targets in the well-studied yeast S. cerevisiae. We compiled a catalog of known and predicted S. cerevisiae *cis*-regulatory elements (Dataset S1) in two ways. First, we retrieved 80 known consensus transcription factor-binding sites from the literature, based in part on information summarized on the Yeast Proteasome Database (Costanzo et al. 2001) and the *Saccharomyces* Genome Database (Weng et al. 2003). The majority of these sequences have been experimentally defined. Six others were identified by virtue of their conservation in the 3′ untranslated regions of closely related *Saccharomyces* species (Kellis et al. 2003), and five downstream elements were computationally predicted from mRNA immunoprecipitation experiments (Gerber et al. 2004). In addition to these known consensus sequences, we used the program MEME (Bailey and Elkan 1994) to identify 597 upstream sequence motifs common to groups of predicted coregulated genes (see below). Genes that contained one or more instance of each of these sequences in the 1,000-bp upstream or 500-bp downstream regions were identified as described in Materials and Methods.

We next identified and manually annotated 264 partially redundant groups of genes that are predicted to be coregulated in S. cerevisiae, based on the genes' similarity in expression, physical association with the same transcription factor, or functional relationships (Dataset S2; see Materials and Methods for details). For each gene group, we systematically scored the enrichment of genes that contained each of the putative regulatory elements identified above, compared to all genes in the S. cerevisiae genome that contained that flanking sequence. Of the 80 consensus sequences, 41 were identified as significant by this criterion. Of these significant sequences, 34 were identified in the gene group known to be regulated by that element (Dataset S3), suggesting an upper limit of 17% false-positive identifications. Of the 597 MEME matrices we identified, only 43 were significantly enriched in the gene group that they were identified in (see matrices in Dataset S4). All but four of these matrices were very similar to the consensus element known to regulate those genes (see Materials and Methods for details). Therefore, out of 19,239 motif-gene group comparisons, we recovered 34 consensus sequences and four additional MEME matrices representing known *cis*-regulatory elements (thus 38 of 80 known elements) and four unannotated MEME matrices that may represent novel S. cerevisiae regulatory sequences, for a total of 42 S. cerevisiae *cis*-elements in 35 unique gene groups.

Many of these S. cerevisiae regulatory elements were shown to be conserved in orthologous regulatory regions from four closely related saccharomyces species (Figure 1, orange species) (Cliften et al. 2003; Kellis et al. 2003). However, it was not known whether these elements are conserved in more distantly related species for which the intergenic regions cannot be aligned. To explore this possibility, we reasoned that many genes that are coregulated in S. cerevisiae should also be coregulated in other fungal species, and that functional *cis*-regulatory elements could be identified with the same methods applied to coregulated S. cerevisiae genes. Therefore, for each group of coregulated S. cerevisiae genes, we identified orthologs in each of 13 other fungal genomes using the method of Wall et al. (2003). This method identifies reciprocal BLAST hits between two genomes that span more than 80% of the protein lengths, thereby providing a more conservative list of putative orthologs than a simple BLAST method. The complete set of orthologs is available in Datasets S5–S13.

For each species-specific gene group, we scored the enrichment of genes that contain each of the 80 consensus sequences or examples of the MEME matrices discovered in the orthologous S. cerevisiae genes, as described above. This procedure was performed separately on each species, so that the identification of an enriched sequence in one species was independent of its identification in the other species. Therefore, when a given sequence was enriched in the orthologous gene groups from multiple genomes, we interpreted this to reflect the conservation of the *cis*-regulatory system represented by that element in the corresponding species. It is important to note that we have characterized this conservation at the level of regulatory networks, which does not necessarily imply that the individual elements upstream of each gene have been perfectly conserved (see Discussion).

### Many* S. cerevisiae Cis*-Regulatory Systems Are Conserved in Other Fungi

**Figure 2:**
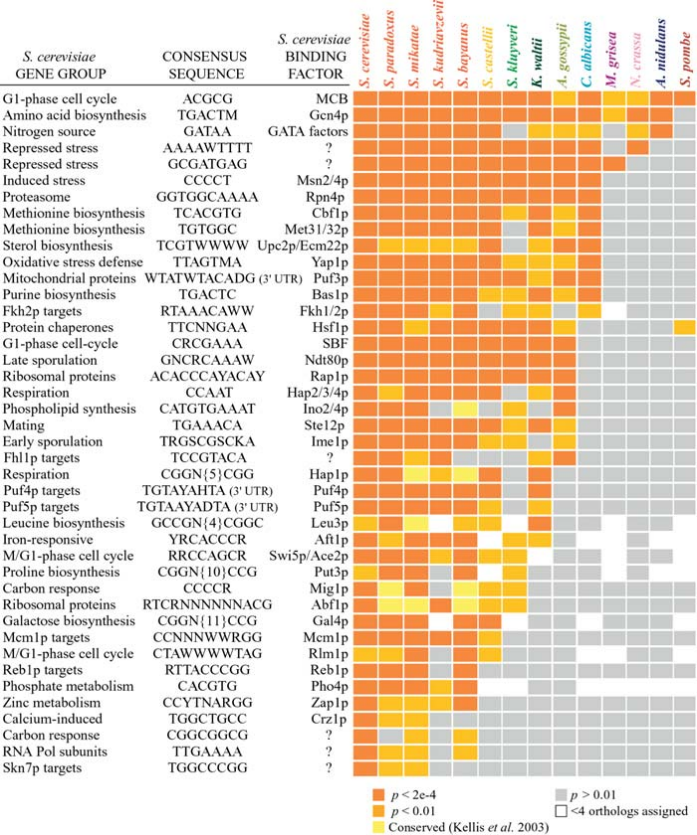
Conservation of*Cis*-Sequence Enrichment in Specific Gene Groups Gene groups from each of the 14 species that are enriched for genes whose flanking regions contain known or novel*cis*-sequences are represented by orange boxes. Each row represents a group of coexpressed S. cerevisiae genes and a single*cis*-regulatory element known or predicted to control the genes' expression, as indicated to the left of the figure. Each column in the figure represents the orthologous gene groups in 14 different fungal species. An orange box indicates that the *S. cerevisiae cis*-regulatory sequence listed to the left of the diagram is enriched in the denoted S. cerevisiae genes or their orthologs in each fungal genome, according to the key at the bottom of the figure. The *p*-values for each group are available in Datasets S14–S46, and the number of orthologs in each gene group is available in Dataset S49. Some*cis*-regulatory elements did not meet our significance cutoff for enrichment but had been previously identified as conserved in related gene groups from the closely related saccharomycete species (Kellis et al. 2003), and these are denoted with a yellow box. A gray box indicates that the denoted sequence was not significantly enriched in that gene group, while a white box indicates that fewer than four orthologs were identified in the species. The rows are organized in decreasing order of the number of species in which the element was enriched.

The patterns of *cis*-sequence enrichment in gene groups from each species strongly suggest that many of the genes coregulated in S. cerevisiae are also coregulated in the other fungal species. Furthermore, these patterns suggest that the expression of those genes is likely to be governed by the same *cis*-regulatory systems. Figure 2 shows the enrichment measured for each *S. cerevisiae cis*-regulatory element in the gene group it is proposed to regulate (represented by each row of the figure) in the 14 fungal species (shown in each column in the figure). (All *p*-values are available in Datasets S14–S46.) All of the 42 elements were identified in the same gene groups from at least three of the four closely related saccharomycete species. The majority of these elements were identified in the orthologous genes from other hemiascomycete species as well: 31 (74%) were identified in *S. castellii,* 23 (56%) and 27 (64%) were found in the related species S. kluyveri and *Kluyveromyces waltii,* respectively, and 21 (50%) and 14 (33%) were found in Ashbya gossypii and *Candida albicans,* respectively. Outside of the hemiascomycete group, we identified three to four (7%–10%) of these elements in the euascomycete fungi and two (5%) in Schizosaccharomyces pombe. Notably, when an identical procedure was performed using randomized consensus sequences, zero sequences were enriched with *p* < 0.0002 in their respective gene group from any species (Figures S1 and S2).

The number of regulatory systems that could be found in each species roughly correlates with the species tree, in that more*cis*-regulatory elements were identified in species closely related to S. cerevisiae compared to the more distantly related fungi. This result could arise from the decreased accuracy of ortholog assignment in the distantly related species, which would hinder the identification of conserved regulatory systems. However, control experiments indicate that our ability to identify each regulatory element by enrichment is largely insensitive to noise in each gene group and to the ortholog assignment parameters (Figure S3 and unpublished data). These results therefore suggest that the number of regulatory systems conserved across species correlates with their divergence times.

A handful of these *cis*-regulatory systems are conserved in all or nearly all of the fungal genomes. For example, the group of G1-phase cell-cycle genes from all species was significantly enriched for genes containing the upstream Mlu1-cell cycle box (MCB) (McIntosh 1993). This sequence regulates the expression of the G1-phase genes from S. cerevisiae (Moll et al. 1992) as well as its distant relative *Sch. pombe* (Lowndes et al. 1992; Malhotra et al. 1993), strongly suggesting that the element has a similar role in the other fungi. Likewise, the Gcn4p binding site was identified in the amino acid-biosynthesis genes from all but *Sch. pombe,* consistent with the known involvement of Gcn4p-like transcription factors in the amino acid-starvation responses of *S. cerevisiae, C. albicans, Neurospora crassa,* and Aspergillus nidulans (Hinnebusch 1986; Ebbole et al. 1991; Tazebay et al. 1997; Tripathi et al. 2002). The expression of nitrogen-catabolism genes in *C. albicans, N. crassa,* and *As. nidulans* is thought to be governed by GATA-like factors (Kudla et al. 1990; Chiang et al. 1994; Marzluf 1997; Limjindaporn et al. 2003), as it is in S. cerevisiae (Magasanik and Kaiser 2002), consistent with our ability to detect upstream GATA-binding elements in the group of nitrogen catabolism genes from these species. In the majority of cases (approximately 80%) in which a given*cis*-regulatory element was identified by enrichment, we could also identify in that species an ortholog of its binding protein from S. cerevisiae. Therefore, the most parsimonious model is that gene-expression regulation through the identified*cis*-regulatory sequence is governed by the orthologous transcription factor in each species.

### Novel Sequences Are Enriched in Coregulated Gene Groups from Other Fungi

In many cases, we were unable to detect significant enrichment of the S. cerevisiae upstream elements in the orthologous gene groups from other species, particularly in the more distantly related fungi. One possible explanation for this observation is that, although the genes are still coregulated in these species, the*cis*-regulatory mechanisms that control their expression have evolved. We therefore searched the upstream regions from each group of orthologous genes for novel sequence motifs, using the program MEME (Bailey and Elkan 1994) and selected matrices that were significantly enriched in the gene group in which they were identified (see Materials and Methods for details). As has been previously noted for this type of motif discovery (Tavazoie et al. 1999; McGuire et al. 2000), the majority of the identified motifs were not significantly enriched in the appropriate gene group and may represent background sequences that are not functional. Thus, a total of 53 matrices were identified as significant in at least one species based on this criterion (the complete list of matrices and enrichment *p* values are available in Datasets S47 and S48). Over half of these were similar to known S. cerevisiae elements shown in Figure 2 and were enriched in the orthologous S. cerevisiae genes. Of the remaining motifs, two recognizably similar matrices were identified in the same gene group from multiple species, suggesting that they represent conserved regulatory systems not present in S. cerevisiae. To further examine this possibility, we scored the enrichment of genes containing examples of the 53 matrices in the orthologous gene groups from all species.

**Figure 3:**
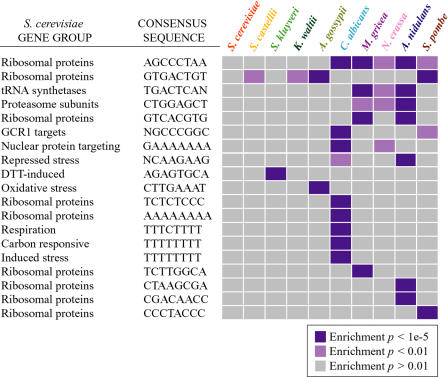
Enrichment of Novel Sequences in Coregulated Genes from Other Species Gene groups from each of the 14 species that are enriched for genes containing novel upstream sequences identified by MEME (see Materials and Methods for details) are shown, as described in Figure 2. Enrichment of genes that contain the*cis*-sequence listed to the left of the diagram is indicated by a purple box, according to the key at the bottom of the figure.

This procedure identified 19 unique MEME matrices that were not identified in the S. cerevisiae genes and therefore may represent novel*cis*-regulatory elements in these fungi (Figure 3). More than a third of these elements were also enriched in the same gene group from other species, providing additional support for their functional relevance. For example, a number of upstream sequences identified in ribosomal-protein genes were enriched in the same gene group from four or five other species, but not from S. cerevisiae. Similarly, sequences identified upstream of tRNA synthetase genes and upstream of the proteasome genes were identified in the same genes from all of the euascomycete fungi *(N. crassa, Magnaporthe grisea,* and *As. nidulans).* In the case of the proteasome genes, MEME identified the same motif upstream of orthologous genes from the related euascomycete *Histoplasma capsulatum,* for which partial genome sequence is available ( http://www.genome.wustl.edu/projects/hcapsulatum/) (unpublished data). That these sequences were identified in the same gene groups from multiple euascomycetes (but not the other species) implies that they are clade-specific. Although future experiments will be required to elucidate the exact roles of these sequences, our observations suggest that the identified *cis*-sequences are functionally relevant and conserved across species.

### *Cis*-Regulatory Element Positions and Spacing Are Also Conserved across Species

The physical locations of many characterized S. cerevisiae *cis*-regulatory elements are restricted to a narrow region upstream of their target genes (Mannhaupt et al. 1999; Tavazoie et al. 1999; McGuire et al. 2000; Lieb et al. 2001; Natarajan et al. 2001). This suggests that these elements must be positioned in the appropriate window of the upstream sequences, perhaps to promote proper interactions between the element's binding protein and other factors (such as nucleosomes or RNA polymerase subunits) (Workman and Kingston 1992; Vashee and Kodadek 1995; Fry et al. 1997; Fry and Farnham 1999; GuhaThakurta and Stormo 2001).

**Figure 4:**
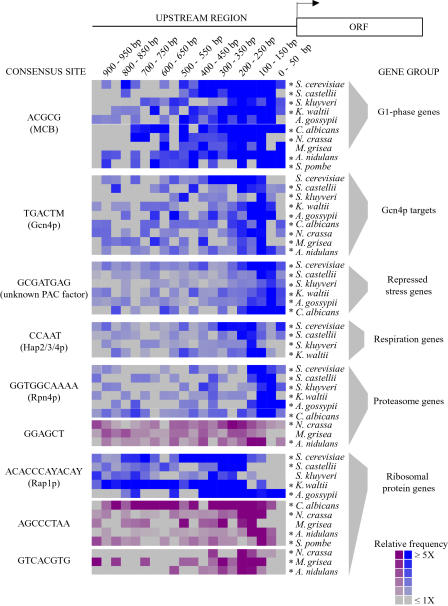
Distribution of *Cis*-Regulatory Elements Upstream of Coregulated Genes The distribution of nine different sequences motifs (represented to the left of the figure by the consensus sequences and their known binding proteins) was measured in 50-bp windows within 1,000 bp upstream of the putative target genes (denoted to the right of the figure). Each colored box represents the frequency of an element in a 50-bp window upstream of the target genes compared to the element's frequency in the corresponding window of all upstream regions in each genome. Blue boxes represent sequences that matched the S. cerevisiae MEME matrices, while purple boxes represent sequences that matched the designated species-specific MEME matrices. Distributions that were significantly different from background in at least one 50-bp window (*p* < 0.01) were identified using the hypergeometric distribution (as described in Materials and Methods) and are denoted by an asterisk.

To characterize the upstream positions of *cis*-regulatory elements in *S. cerevisiae,* we compared the fraction of elements in 50-bp windows upstream of their target genes to the fraction of elements in the same 50-bp window upstream of all genes in the S. cerevisiae genome. (This model is required to overcome the nonrandom nucleotide distribution immediately upstream of genes in this and other species, as described in Materials and Methods.) We found that many of the *S. cerevisiae cis*-regulatory elements are nonrandomly distributed upstream of their target genes (Figure 4, blue boxes). Each element shows a different window of peak enrichment in S. cerevisiae. This likely reflects mechanistic differences between the regulatory systems that control the expression of each set of genes.

In the majority of cases, when a *cis*-regulatory system was conserved in another species, the corresponding element had a similar upstream distribution to that seen in *S. cerevisiae,* in that the distributions had the same window of peak enrichment (Figure 4). This is significant, as the underlying genomic distribution of many of these sequences is substantially different in each species, due in part to the different GC content of some of the genomes (unpublished data). For many regulatory systems, there was no correlation between the positions of individual elements in orthologous upstream regions from multiple species (although there were some exceptions; Figures S4 and S5). This indicates that the distributions of these elements have been conserved, even though the precise positions of individual elements have not (see Discussion). In addition to the conserved S. cerevisiae elements, many of the novel*cis*-sequences presented in Figure 3 also showed nonrandom distributions in the species in which they were identified (Figure 4, purple boxes). Thus, the positional distribution of *cis*-regulatory elements appears to be a general feature of *cis*-regulation in multiple ascomycete species.

**Figure 5:**
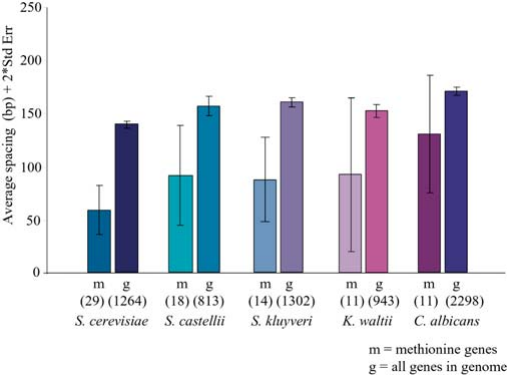
Spatial Relationships between*Cis*-Regulatory Elements The mean spacing between the Cbf1p- and Met31/32p- binding sites within 500 bp upstream of the methionine biosynthesis genes (m) and of all of the genes in each genome (g) was calculated for the species indicated. The error bars represent twice the standard error, indicating the range of the estimated means with 95% confidence. The values below each plot indicate the number of binding-site pairs used in each calculation.

In one case, the close spacing between two*cis*-regulatory elements was conserved across species. Chiang et al. (2003) previously reported that the distance between the Cbf1p- and Met31/32p-binding sites upstream of the methionine biosynthesis genes is closer than expected by chance. We found this feature to be conserved in other species as well. The Cbf1p and Met31/32p elements were independently identified upstream of the methionine genes from almost all of the hemiascomycetes (see Figure 2). In addition, the closer-than-expected spacing between these sequences was also conserved in these species (Figure 5). The spacing between elements was independent of the exact positions of the Cbf1p or Met31/32p sites in the saccharomycete species, indicated by permutation tests performed as previously described (*p* < 0.05; Chiang et al. 2003). Thus, the close spacing between these sites is not simply due to the conserved positioning of the individual elements in each orthologous upstream region, but likely resulted from an evolutionary constraint on the distance between these sequences (see Discussion).

### Evolution of the Proteasome *Cis*-Regulatory Element in S. cerevisiae and C. albicans

We were particularly interested in exploring patterns of *cis*-element evolution across fungi. One interesting example is the case of Rpn4p, a nonclassical Cys2-His2 zinc-finger protein known to regulate proteasome gene expression in S. cerevisiae (Mannhaupt et al. 1999; Xie and Varshavsky 2001). For the group of S. cerevisiae proteasome genes, the enrichment of genes containing the known Rpn4p binding site was highly significant (GGTGGCAA; *p* < 6 × 10^–41^). The same consensus sequence was also enriched in the orthologous upstream regions of all of the hemiascomycete fungi, but not in the upstream regions retrieved from fungi outside of the hemiascomycete group. We noticed that, in addition to the Rpn4p consensus site, a number of related hexameric sequences were also highly enriched in the orthologous upstream regions from C. albicans (unpublished data). This hinted at the possibility that a slightly different set of regulatory sequences governs the expression of the C. albicans proteasome genes.

**Figure 6:**
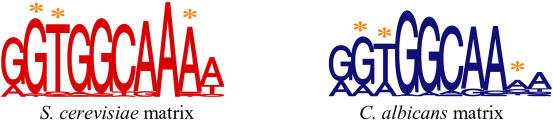
Position-Weight Matrices Representing Proteasome C*is*-Regulatory Elements Sequences within 500 bp upstream of the S. cerevisiae or C. albicans proteasome genes that matched the species-independent meta-matrix were identified as described. The identified sequences were used to generate sequence logos (Crooks et al. 2004) to represent the set of *cis*-sequences from S. cerevisiae (left) or from C. albicans (right). The height of each letter represents the frequency of that base in that position of the matrix. Positions in the matrices that are statistically different (see Materials and Methods for details) are indicated with an asterisk.

To further explore this possibility, we compared sequences found upstream of the proteasome genes from S. cerevisiae and C. albicans. To identify these sequences in an unbiased way, we first generated a species-independent “meta-matrix” based on a limited subset of the proteasome upstream regions from both species (see Materials and Methods for details). We then identified all examples of the meta-matrix upstream of the proteasome genes from S. cerevisiae and *C. albicans,* partitioned the sequences according to their species, and calculated two species-specific position-weight matrices (Figure 6). These matrices were statistically different at the second, third, and ninth positions (*p* < 0.01; see Materials and Methods for details) and indicated that the C. albicans matrix had less basepair specificity at these positions.

The matrices are useful because they summarize the set of related sequences that are common to the upstream regions in each group, but a more direct assessment of these elements is to inspect the sequences directly. Sequences upstream of the S. cerevisiae and C. albicans proteasome genes that matched the “meta-matrix” described above were combined and organized by sequence similarity, using a hierarchical clustering method described in Materials and Methods. The sequences could be classified into three general categories (Figure S6). The first category consisted of related sequences that were found in both S. cerevisiae and C. albicans proteasome upstream regions, the second was composed of sequences found almost exclusively upstream of S. cerevisiae genes, and the third was composed of elements found only upstream of the C. albicans proteasome genes. Manual inspection of the proteasome-gene upstream regions supported these classifications: There were zero instances of the S. cerevisiae-specific 10-mer GGTGGCAAAW upstream of any C. albicans proteasome genes, although nearly 75% of the S. cerevisiae proteasome genes contained this upstream sequence. Similarly, zero instances of the C. albicans-specific 10-mer GRAGGCAAAA were found upstream of S. cerevisiae proteasome genes, whereas 25% of the C. albicans genes contained the element. These observations suggest that S. cerevisiae and C. albicans use different sequences to govern the expression of the proteasome genes.

### Sc_Rpn4p and Ca_Rpn4p Have Different In Vitro Binding Specificities

Two mutually exclusive possibilities could explain the differences in the upstream sequences found in S. cerevisiae and C. albicans proteasome genes. One model is that the species-specific differences in these*cis*-sequences reflect differences in the binding specificity of S. cerevisiae Rpn4p and its ortholog in C. albicans. Alternatively, the two transcription factors may bind with the same specificity, indicating that some other feature(s) contributed to the differences in these sets of sequences. Examination of the nucleotide frequencies in each genome ruled out the possibility that the differences in *cis*-sequences arose simply by drift in the underlying genomic base composition (unpublished data). To further distinguish between the above models, we cloned and purified S. cerevisiae Rpn4p (Sc_Rpn4p) and the orthologous protein from C. albicans (Ca_Rpn4p) and measured their binding properties in vitro. The interaction of each protein with three different DNA sequences (each representing one of the three classes of upstream sequences described above) was measured using the Biacore 3000 affinity system, which measures biomolecular interactions between proteins and DNA (see Materials and Methods for details). Briefly, double-stranded DNA fragments containing the relevant sequences were immobilized onto a solid surface, and real-time protein-DNA interactions were measured as each protein was passed over the immobilized DNAs and allowed to bind (reviewed in Malmqvist 1999).

**Figure 7:**
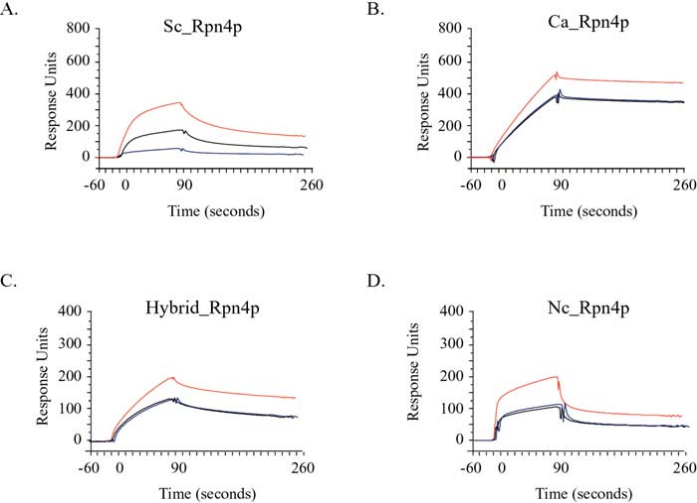
In Vitro DNA-Binding Profiles of Rpn4p Proteins Profiles of 50 nM Sc_Rpn4p (A), Ca_Rpn4p (B), Hybrid_Rpn4p (C), and Nc_Rpn4p (D) binding to Sequence A (S. cerevisiae-specific; red curve), Sequence B (C. albicans-specific; blue curve), and Sequence C (hybrid; black curve) are shown. Protein was injected into the Biacore system at time = 0 for a duration of 90 sec, after which time buffer was injected and the protein dissociated from the Biacore chip. The scale of each binding profile was adjusted such that the binding levels to Sequence A are comparable for all species.

The results of these in vitro binding experiments revealed that Sc_Rpn4p and its ortholog Ca_Rpn4p have different DNA-binding specificities. Figure 7 shows the binding of Sc_Rpn4p and Ca_Rpn4p to the S. cerevisiae-specific Sequence A (GGTGGCAAAA), the C. albicans-specific Sequence B (GAAGGCAAAA), and Sequence C (AGTGGCAACA), which represents sequences found in both species. Sc_ Rpn4p bound preferentially to Sequence A and, to a lesser extent, to Sequence C; however, the binding of Sc_Rpn4p to Sequence B was barely detectable (Figure 7A). Ca_Rpn4p also bound preferentially to Sequence A, but in contrast to Sc_Rpn4p, this protein bound nearly indistinguishably to Sequence B and Sequence C in vitro (Figure 7B).

**Figure 8:**
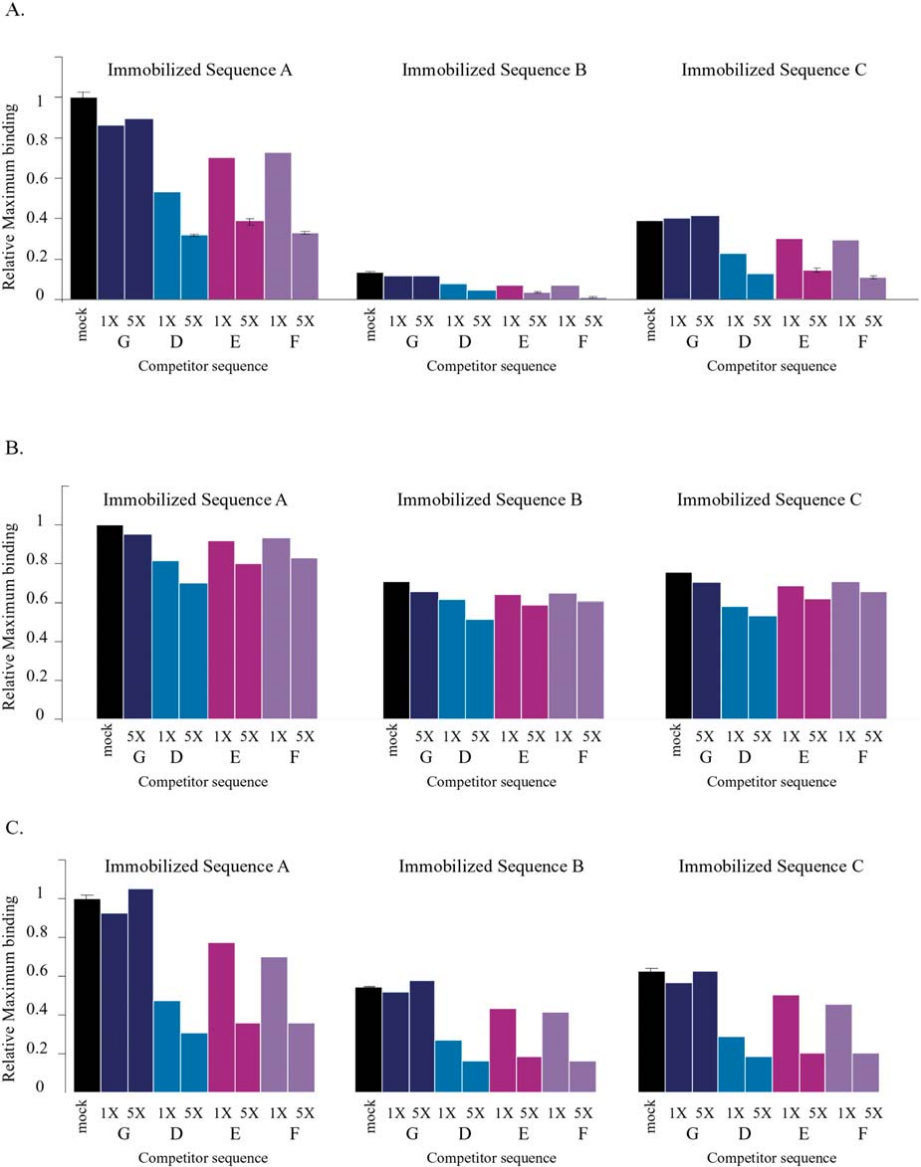
In Vitro Competition for DNA Binding The maximum response units of binding were measured for Sc_Rpn4p (A), Ca_Rpn4p (B), or the hybrid protein (C) binding to Sequence A (left graphs), Sequence B (center graphs), and Sequence C (right graphs) in the absence (“mock”) or presence of a 1× or 5× molar excess of competitor fragments: Sequence G (with a core sequence of CTGCATTTGG), Sequence D (GGTGGCAAAA), Sequence E (AGTGGCAAAA), and Sequence F (GGTGGCAACA). Each histogram shows the maximum response units of binding, relative to the maximum response units measured for that protein binding to the Sequence A in the absence of competitor. Replicate experiments were performed for each mock reaction and the 5:1 competition experiments for Sc_Rpn4p protein. The range of replicate measurements was very narrow and is indicated by the error bars.

In all cases, the DNA binding was specific, as competitor fragments that were similar to the Sc_Rpn4p consensus sequence, but not a dissimilar control fragment, were effective inhibitors of binding when preincubated with the protein (Figure 8). This was true even for Sc_Rpn4p binding to Sequence B, despite the low levels of binding to this sequence. A fragment identical to the immobilized Sequence A was the best competitor for both Sc_Rpn4p and Ca_Rpn4p binding to all immobilized sequences, compared to competitor fragments with single basepair differences in either the first or ninth position of the element. This was surprising in the case of Ca_Rpn4p, since the lower basepair specificity in the ninth position of the C. albicans proteasome matrix (see Figure 6) predicted that sequence variation at this position would not significantly affect binding.

A reasonable expectation is that amino acid differences in the DNA-binding domains of each protein account for the differences in their specificity, perhaps by promoting subtly different contacts between each protein and its DNA substrate. While this is not an obligate explanation, we found it to be the case: A hybrid protein that consisted of the amino-terminal portion of Sc_Rpn4p fused to the carboxyl-terminal DNA-binding domain of Ca_Rpn4p (see Materials and Methods for details) was able to bind Sequence B indistinguishably from Sequence C, as did the native Ca_Rpn4p (see Figure 7C). Again, the binding was specific, since the expected sequences, but not the negative control, were able to compete for binding (Figure 8). These results reveal that amino acid differences between the Sc_Rpn4p and Ca_Rpn4p DNA-binding domains account for the altered specificity of these proteins.

### Nc_Rpn4p Has the Same In Vitro Specificity as Ca_Rpn4p

Although we could not identify Rpn4p-like elements upstream of the majority of proteasome genes from the other fungi, we did identify a different sequence, GGAGCT, upstream of the proteasome genes from the euascomycete fungi. Because each of these fungi has an ortholog of Rpn4p, we cloned Nc_Rpn4p as a representative and characterized its binding to the novel sequence and to Sequence A, B, and C described above (see Materials and Methods for details). Nc_Rpn4p did not bind detectably to the GGAGCT sequence in vitro, similar to its orthologs Sc_Rpn4p and Ca_Rpn4p that did not bind this sequence (unpublished data). In contrast, Nc_Rpn4p bound to the Rpn4p-like elements with a binding profile similar to Ca_Rpn4p: Nc_Rpn4p bound maximally to Sequence A and bound nearly identically to Sequence B and Sequence C on the Biacore chip (see Figure 7D). Since the majority of proteasome genes from the euascomycete fungi do not contain these sequences, these results suggest that Nc_Rpn4p does not regulate proteasome gene expression.

## Discussion

The ascomycete fungi represent nearly 75% of all fungal species, and their diversity is evident by their unique morphologies, life styles, environmental interactions, and niches (Ainsworth et al. 2001). This diversity has been shaped by over a billion years of evolution (Berbee and Taylor 1993; Heckman et al. 2001) and has almost certainly been affected by variation in gene expression. To explore the evolution of gene-expression regulation in these fungi, we have examined the *cis*-regulatory networks of 14 ascomycete species whose genomes have been sequenced, using a framework that is not dependent on multiple alignments of orthologous regulatory regions. We have identified probable *cis*-acting sequences in each of these species by applying motif search and discovery methods to the flanking regions of orthologs of coregulated S. cerevisiae genes. Our ability to identify such sequences in the same gene groups from multiple species strongly suggests that the coregulation of those genes has been conserved. Examples from our analysis indicate that in many cases the genes' coregulation is governed by a conserved regulatory system, while other examples suggest that some regulatory networks have evolved. These examples provide insights into the functional constraints that underlie the evolution of gene-expression regulation, as summarized below.

### Conservation of *Cis*-Regulatory Systems

Our results indicate that a large number of *cis*-regulatory networks that function in S. cerevisiae are conserved in other ascomycete species. This is expected for the closely related species, since conserved regulatory elements can be readily identified in alignments of orthologous regulatory regions (Cliften et al. 2003; Kellis et al. 2003). However, we show here that many of the *cis*-regulatory systems represented by these elements are conserved over much longer evolutionary time frames, beyond those for which orthologous noncoding regions can be aligned. For example, 50%–75% of the regulatory systems identified in S. cerevisiae are also found in S. kluyveri and *S. castellii,* which are diverged enough from S. cerevisiae that much of the gene synteny is lost and most orthologous intergenic regions cannot be aligned (Cliften et al. 2003). Over a third of these regulatory systems were identified in *C. albicans,* which is estimated to have diverged from S. cerevisiae over 200 million years ago, and a small number of regulatory networks have been conserved since the origin of the Ascomycetes some 500 million to a billion years ago (Berbee and Taylor 1993; Heckman et al. 2001). It is likely that we have underestimated the number of conserved regulatory networks, partly because of statistical limitations of our method. Nonetheless, these data indicate that regulatory networks can be conserved over very long periods of evolution.

Despite the widespread conservation of *cis*-regulatory networks, it is important to note that this does not necessarily imply that the individual *cis*-elements have remained perfectly conserved. For example, while we could identify the same *cis*-sequences in orthologous gene groups, the positions of the individual elements in orthologous upstream regions in many cases appear to have changed (see Figure S4). Evolution of *cis*-element position has been observed in closely related drosophilids, mammals, and other species (Ludwig and Kreitman 1995; Ludwig et al. 1998; Piano et al. 1999; Dermitzakis and Clark 2002; Scemama et al. 2002; Dermitzakis et al. 2003) and is proposed to occur by two general mechanisms (reviewed in Wray et al. 2003). The first is binding-site turnover, whereby the appearance of a new *cis*-element elsewhere in a promoter can compensate for the loss of a functional element in the same regulatory region. Simulation studies show that *cis*-element turnover occurs frequently over short evolutionary time scales and is likely to play an important role in gene-expression regulation (Stone and Wray 2001; Dermitzakis et al. 2003). Alternatively, small insertions and deletions in a regulatory region can permute the *cis*-element's position without changing the element's sequence (Ludwig and Kreitman 1995; Piano et al. 1999; Ruvinsky and Ruvkun 2003). Thus, regulatory regions appear to be relatively plastic in their organization. Despite this plasticity, however, a gene's expression pattern and the regulatory system governing its expression can remain intact even though the gene's flanking regulatory region has undergone reorganization (Piano et al. 1999; Ludwig et al. 2000; Scemama et al. 2002; Hinman et al. 2003; Romano and Wray 2003; Ruvinsky and Ruvkun 2003). This indicates that some combination of purifying selection and drift (Ludwig et al. 2000) can act to maintain the appropriate regulatory connections to conserve the gene's expression pattern.

Although the positions of many of the individual *cis*-elements have evolved in these species, we found that the distribution of elements upstream of their gene targets was often similar across species. This suggests that there has been constraint on the region in which the elements are positioned, without pressure to maintain the exact positions of individual elements. One explanation for this model is that mechanistic features of these regulatory systems are also conserved across species (Wray et al. 2003). For example, the restricted location of *cis*-regulatory elements may promote interactions between the cognate binding protein and other regulatory proteins. Therefore, selective pressure may act to maintain these interactions through the relative positions of the underlying binding sites. This model may also explain the conserved close spacing between Cbf1p and Met31/32p elements in methionine biosynthesis genes from the hemiascomycete fungi. These transcription factors are proposed to act cooperatively in S. cerevisiae to recruit additional transcriptional regulators (Blaiseau and Thomas 1998). That the spacing between the Cbf1p and Met31/32p elements is closer than expected in other species as well suggests that the cooperative interaction between the factors has been conserved across the Hemiascomycetes.

### Evolution of *Cis*-Regulatory Networks

In addition to the clear cases of network conservation discussed above, we also found evidence for the evolution of *cis*-regulatory systems. Our ability to identify novel sequences enriched in orthologs of coregulated S. cerevisiae genes implies that, although the genes are still coregulated in those species, the systems governing their expression have changed. This indicates that the regulatory regions of those genes coevolved to contain the same *cis*-sequences.

We were interested in identifying global predictors of the relative rates of *cis*-regulatory network evolution, but these factors remain enigmatic. Unlike the evolutionary rates of protein coding regions, for which essential proteins typically evolve at a slower rate (Wilson et al. 1977; Hirsh and Fraser 2001; Krylov et al. 2003; H. B. F., personal communication), we found no evidence for a retarded rate of evolution/loss of the *cis*-regulatory systems of essential genes (unpublished data). For example, the proteasome subunits and the ribosomal proteins are among the most highly conserved proteins, and the genes that encode them are expressed with similar patterns in *S. cerevisiae, C. albicans,* and *Sch. pombe* (Gasch et al. 2000; Chen et al. 2003; Enjalbert et al. 2003). Nonetheless, we identified different upstream sequences for these groups in the different species we analyzed, suggesting that the regulation of the genes' expression has evolved even though their expression patterns have not. This is consistent with previous observations of developmentally regulated genes in higher organisms, whose temporal and spatial expression can be conserved across taxa despite divergence in their regulation (Takahashi et al. 1999; True and Haag 2001; Scemama et al. 2002; Hinman et al. 2003; Romano and Wray 2003; Ruvinsky and Ruvkun 2003; Wang et al. 2004). In contrast, we observed that proteins involved in mating have a high rate of evolution, yet we could identify the Ste12p binding site (Fields and Herskowitz 1985) upstream of mating genes in nearly all of the hemiascomycetes. Consistently, orthologs of Ste12p are known to be required for mating in distantly related fungi that mate through significantly different processes (Lengeler et al. 2000; Vallim et al. 2000; Young et al. 2000; Chang et al. 2001). Since mating may be triggered by similar environmental cues (Lengeler et al. 2000), evolutionary pressure may have conserved the regulatory system that mediates this process (to the extent of our observations), even though the mating proteins have evolved.

Although we could not find global correlates with the patterns of *cis*-regulatory network evolution, a number of individual examples from our analysis are consistent with specific models of network evolution. These examples are discussed below.

### Addition of Gene Targets into an Existing Regulatory Network

Sequences that match *cis*-regulatory elements can readily appear in noncoding DNA through drift. In the same way that this process can promote binding site turnover within a given regulatory region, it can create de novo elements in the regulatory regions of random genes, giving rise to novel targets of that regulatory system (Stone and Wray 2001; Rockman and Wray 2002). The addition of novel targets into *cis*-regulatory systems may have occurred in the case of E2F-like transcription factors. In S. cerevisiae, the related MCB (ACGCG) and Swi4-Swi6 cell-cycle box, or SCB (CGCGAAA) regulatory elements are found upstream of G1-phase cell-cycle genes, similar to the E2F element found in these genes in worms, flies, humans, and plants (Lowndes et al. 1992; Malhotra et al. 1993; DeGregori 2002; Ren et al. 2002; De Veylder et al. 2003; Rustici et al. 2004). What is striking about the conservation of this network is that cell-cycle progression is markedly different in these organisms: The hemiascomycete fungi replicate by budding, unlike the filamentous fungi in the euascomycete group, the fission yeast *Sch. pombe*, and the other higher eukaryotes. While some of the genes regulated by these elements are well conserved across organisms (namely, the DNA replication proteins), genes whose products are involved in budding are also expressed in G1 phase and regulated by these elements in S. cerevisiae (Spellman et al. 1998; Iyer et al. 2001) and likely in its budding cousins as well. Because these genes are not conserved outside the hemiascomycete clade, and since it is unlikely that budding represents the ancestral mode of replication, this suggests that genes involved in budding were assumed into an existing *cis*-regulatory network in these yeasts.

### Coevolution of an Existing Regulatory Network

Mutation of a *cis*-regulatory element can be compensated by the stabilizing effects of binding site turnover (Ludwig et al. 2000), as discussed above, but it could also be overcome by corresponding changes in its DNA-binding protein, such that the interaction between the two is maintained. Parallel changes in DNA element and protein sequence can occur to conserve the overall regulatory network (i.e., the same binding protein regulating the same set of genes), despite evolution of their molecular interaction. We found slightly different sets of sequences enriched upstream of the proteasome genes from S. cerevisiae versus *C. albicans,* and these differences corresponded with the different binding specificities of Sc_Rpn4p and Ca_Rpn4p in vitro. This result is consistent with the model that the binding specificity of Sc_Rpn4p and Ca_Rpn4p coevolved with the elements found upstream of the proteasome genes in each species.

Neither Ca_Rpn4p nor the hybrid protein functioned in an in vivo reporter system (unpublished data); however, Sc_Rpn4p could transcribe a reporter gene to higher levels if Sequence A was present in its promoter compared to when Sequence B or a minimal promoter was placed upstream of the reporter gene (see Figure S7). These results are consistent with the hypothesis that Sc_Rpn4p ineffectively initiates transcription from the C. albicans-specific element. Since Ca_Rpn4p and Nc_Rpn4p both bind significantly to Sequence B, it is likely that this was also true of the proteins' common ancestor and that Sc_Rpn4p largely lost the ability to bind productively to this sequence.

**Figure 9:**
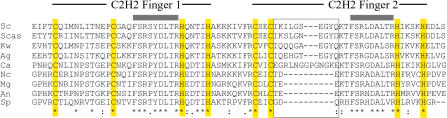
Sequence Alignment of the DNA-Binding Domain of Rpn4p and Its Orthologs Clustal W was used to identify a multiple alignment between S. cerevisiae Rpn4p and its orthologs in the other fungi; the alignment over the DNA binding domain is shown. No ortholog was identified by our method in *S. kluyveri,* apparently due to poor sequence coverage in that region (unpublished data). The conserved cysteine and histidine residues of the two C2H2 zinc-finger domains are highlighted in yellow, and the domain in each finger that is predicted to contact the DNA is indicated with a gray bar. The region of sequence variation between the hemiascomycete and euascomycete Rpn4p proteins is indicated with a box.

The altered specificity of Sc_Rpn4p is due to amino acid differences in its DNA-binding domain, since the hybrid Rpn4p (containing the Ca_Rpn4p DNA binding domain) bound to Sequence B as well as it did to Sequence C (see Figure 7C). Determining which residues are responsible for the altered activity is a difficult task, however, since all of the residues known to participate in zinc coordination and DNA contact (Rhodes et al. 1996; Wolfe et al. 1999; Wolfe et al. 2000; Pabo et al. 2001; Benos et al. 2002) are perfectly conserved between these orthologs (Figure 9). One obvious difference in the orthologous proteins is the spacing between the cysteine and histidine pair in the second zinc finger, which is proposed to contact the first half of the DNA-binding site (Wolfe et al. 2000; Pabo et al. 2001) wherein the base-specificity differences reside. Sc_Rpn4p, Ca_Rpn4p, and the euascomycete Rpn4p orthologs all vary in amino acid length and identity in this region, which implicated the region as relevant to the specificity differences. However, a mutant Sc_Rpn4p that contained the Nc_Rpn4p sequence in this region (see Figure 9) had the same binding specificity as the wild-type Sc_Rpn4p (albeit with less activity; unpublished data), indicating that this region alone is not sufficient to explain the differences in binding profiles.

### Cooption of a Regulatory System to Govern a Different Set of Genes

An extreme example of the previously discussed modes of evolution is the complete alteration of a regulatory system's target genes (True and Carroll 2002). This may have occurred for the Rpn4p regulatory system sometime after the divergence of the euascomycete and hemiascomycete fungi. Our data suggest that, while Sc_Rpn4p and Ca_Rpn4p control proteasome-gene expression in these species, the euascomycete orthologs of this transcription factor probably do not. Nc_Rpn4p did not bind the novel sequence we identified upstream of euascomycete proteasome genes, and reciprocally the majority of these genes did not contain examples of the Rpn4p binding site. One possibility is that Nc_Rpn4p and its orthologs regulate a different set of genes in the euascomycete clade. Preliminary investigation of orthologous euascomycete genes that contain examples of the Ca_Rpn4p matrix (used as a surrogate for the Nc_Rpn4p binding matrix) did not reveal any obvious relationships in the genes' functional annotations or striking similarities in their patterns of expression (T. Kasuga, personal communication). Interestingly, however, the orthologs of *RPN4* in all three euascomycete species contained upstream Rpn4p elements, raising the possibility that this gene is autoregulated at the level of expression in these fungi. Future experiments will test the function of this factor in N. crassa as well as the role of the novel sequence in mediating proteasome gene expression.

The converse of this situation is that the regulatory regions of coregulated genes must coevolve, such that they all contain the same regulatory elements recognized by the new system. This apparently occurs despite strong constraint on the genes' expression patterns. For example, most proteasome subunits are essential and required in proper stoichiometric amounts (Russell et al. 1999; Kruger et al. 2001). Nonetheless, we found different *cis*-sequences upstream of the proteasome genes from the hemiascomycete and euascomycete fungi. Another example can been seen in the ribosomal protein genes, which must also be expressed to the same relative levels (Warner 1999; Zhao et al. 2003). In all species, we could find elements upstream of the ribosomal proteins, but different *cis*-sequences were identified in subsets of these species (see Figures 2 and 3). How the regulatory systems that control the genes' expression evolve is unclear. This process may involve an intermediate stage in which the genes' expression is controlled by two distinct, but partially redundant, regulatory systems (True and Haag 2001; True and Carroll 2002). Differential loss of one system in two diverged species would render the orthologous genes coregulated by different regulatory systems. This model for regulatory system “turnover” is in direct analogy to the case of binding site turnover, in which partially redundant *cis*-elements that are created by drift coexist in a regulatory region before they are differentially lost in the diverged species (Ludwig et al. 2000; Stone and Wray 2001).

### Conclusions and Future Directions

We have provided a framework for studying *cis*-regulatory evolution without relying on alignments of intergenic regions. The evolutionary dynamics of transcriptional regulation is evident from the examples we have presented. We expect that as more complete fungal genomes emerge, particularly for fungi with intermediate evolutionary relationships, important gaps in the existing phylogeny will be filled. These key species may provide a window into intermediate stages of *cis*-element evolution, allowing us to further delineate the patterns of and constraints on the evolution of *cis*-regulation.

## Materials and Methods

### 

#### Genome sequences

Genome sequence and open reading frame (ORF) annotations for the saccharomycete species were obtained from P. Cliften, M. Kellis, and the *Saccharomyces* Genome Database (Goffeau et al. 1996; Cliften et al. 2003; Kellis et al. 2003). Sequences for other genomes were downloaded from the published or listed Web sites as follows. K. waltii (Kellis et al. 2004), A. gossypii (Dietrich et al. 2004), C. albicans (Assembly 6; http://www-sequence.stanford.edu/group/candida/) (Jones et al. 2004), N. crassa (Release 3; Galagan et al. 2003), M. grisea (Release 2; http://www-genome.wi.mit.edu/annotation/fungi/magnaporthe/) , *As. nidulans* (Release 3.1; http://www.broad.mit.edu/annotation/fungi/aspergillus/) , and *Sch. pombe* (Wood et al. 2002). A conservative list of putative ORFs from *S. kudriavzevii,* *S. castellii,* and S. kluyveri was generated, taking all ORFs of more than 100 amino acids as putative genes. ORFs orthologous to S. cerevisiae genes were identified as described below; some intron-containing S. cerevisiae genes that may also contain introns in these species (namely ribosomal protein genes) were identified by tBLASTn and manually added to the list of orthologs for these species.

Orthologs between S. cerevisiae and *S. paradoxus, S. mikatae,* and S. bayanus (Kellis et al. 2003) were downloaded from the *Saccharomyces* Genome Database ( http://www.yeastgenome.org/) . All other orthologs to S. cerevisiae genes were assigned using the method of Wall et al. (Wall et al. 2003) using a BLAST e-value cutoff of 10^-5^ and the requirement for fewer than 20% gapped positions in the Clustal W alignments. The number of orthologs assigned in each species is listed in Table 1, and the complete results are available in Datasets S5–S12.

**Table 1:**
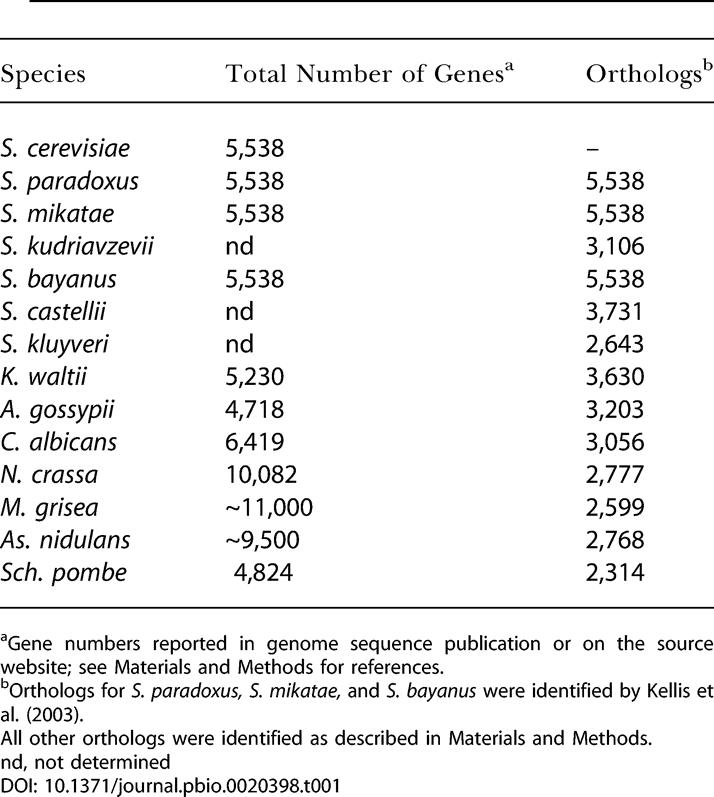
Orthologs Assigned to S. cerevisiae Genes

^a^Gene numbers reported in genome sequence publication or on the source website; see Materials and Methods for references

^b^Orthologs for *S. paradoxus, S. mikatae,* and S. bayanus were identified by Kellis et al. (2003)

All other orthologs were identified as described in Materials and Methods

nd, not determined

#### ***S. cerevisiae*** gene clusters

Groups of known or putatively coregulated genes were identified in three ways. First, we used hierarchical (Eisen et al. 1998) and fuzzy *k*-means (Gasch and Eisen 2002) clustering to organize publicly available yeast gene expression data (DeRisi et al. 1997; Spellman et al. 1998; Gasch et al. 2000; Lyons et al. 2000; Ogawa et al. 2000; Primig et al. 2000; Gasch et al. 2001; Yoshimoto et al. 2002), taking gene clusters that were correlated by more than about 0.7 or with a membership of 0.08 or greater (Gasch and Eisen 2002). Second, we identified genes or transcripts whose flanking regions are physically bound by the same DNA or RNA binding proteins, as indicated by immunoprecipitation experiments (Simon et al. 1993; Iyer et al. 2001; Lieb et al. 2001; Simon et al. 2001; Lee et al. 2002; Gerber et al. 2004): For the DNA immunoprecipitation experiments, genes were ranked according to the published binding *p* values, and a sliding *p* value (between 10^-2^ and 10^-4^) was applied such that at least 20 genes were selected in each group. Transcripts that are bound by RNA binding proteins were taken from (Gerber et al. 2004). Finally, genes with the same functional annotations (Weng et al. 2003), and genes known to be coregulated by various transcription factors (Gasch et al. 2000; Lyons et al. 2000; Ogawa et al. 2000; Shakoury-Elizeh et al. 2004), were grouped together. In all, we identified 264 partially redundant groups of S. cerevisiae genes that are likely to be coregulated. These gene groups ranged in size from four to 570 genes, with a median size of 17 genes per group. The complete gene groups are available in Dataset S2.

#### Motif identification and enrichment

We compiled from the literature a list of 80 known transcription factor-binding sites, represented by IUPAC consensus sequences (Dataset S1) (Costanzo et al. 2001; Weng et al. 2003). Unless otherwise noted, we searched 1,000 bp upstream or 500 bp downstream of the genes from each group in each fungal genome for sequences that matched the consensus binding sites, by doing string comparisons on both strands using PERL scripts. For each group of genes identified above, we scored the enrichment of genes whose flanking regions (either 500 bp upstream, 1,000 bp upstream, or 500 bp downstream) contain one or more example of each *cis*-regulatory element, using the hypergeometric distribution



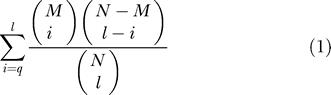



where *M* is the number of genes that contain the motif in a group of *i* selected genes, relative to *N* genes that contain the motif in a genome of *l* genes. A *p* ≤ 0.0002 (approximately 0.01/80 tests) was deemed statistically significant for the consensus sequences, although if the sequence was enriched in the known group of target genes, we relaxed the cutoff to *p* = 0.01. A cutoff of *p* ≤ 2 × 10^–5^ was applied to sequences that matched the MEME matrices. For the Mig1p and GATA binding sequences, which are sufficiently short and occur frequently in each genome, we also scored the enrichment of genes whose upstream region contained two or more examples of the known binding sites.

For each group of genes, we also ran the motif-finding algorithm MEME (Bailey and Elkan 1994) on the upstream regions of S. cerevisiae genes or their orthologs in each species, using a two-component mixture model both with and without a motif-width specification of 8 bp. Unless otherwise noted, we used 500 bp upstream (for the hemiascomycetes) or 1,000 bp upstream (for the euascomycetes and *Sch. pombe*) of the genes in each group. Thus, for each group of coregulated genes, we performed 14 MEME analyses (each identifying three matrices) on the upstream regions of the genes from a given species. Matrices that matched known S. cerevisiae regulatory elements were identified by manual and automated comparisons, similar to that previous described (Hughes et al. 2000). A position-weight matrix was calculated for each motif on the basis of *n* motif examples MEME identified by counting the number of occurrences of each base at each position in *n* motifs, adding one pseudocount, and dividing by *n* + 4. A log-likelihood score *S* was calculated for each motif example as follows.



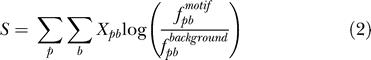



In this formula, *p* is each position in the motif, *b* is the base {GACT} and *X* is a matrix of indicator variables representing the sequence, where *X_pb_* = 1 if the sequence has base *b* at position *p*, and zero otherwise. The probabilities of bases in the motif according to the position-weight matrix are represented by *f ^motif^,* and the probabilities of bases in the genomic background are represented by *f ^background^* (see below). The score *S*′ was assigned to each matrix, equal to 0.75× the average *S* of the motif examples, using the base frequency from each genome as the background model (G/C = 0.2 and A/T = 0.3 for all species except N. crassa, where G/C/A/T = 0.25). This score was used as a cutoff to identify genomic examples of the matrix.

To identify genes whose upstream regions contained examples of each motif, we calculated the log-likelihood *S* of each 8-bp sequence within the 1,000 bp upstream region of each gene. The background model was based on the genomic nucleotide frequency in the 50 bp upstream window corresponding to the position of the sequence being assessed. We used this model to overcome the species-specific positional nucleotide biases immediately upstream of coding sequences (A. M. M., A. P. G., D. Y. C., and M. B. E., unpublished data). A sequence was considered a match to the matrix if *S* > *S* ′. The enrichment of genes that contained each motif was scored using the hypergeometric distribution, as described above. A *p* ≤ 1 × 10^–5^ (0.01 divided by the number of matrices tested in each species) was considered statistically significant. Out of the MEME matrices trained on the non-S. cerevisiae species, 53 were enriched in the gene group in which they were identified. Of these elements, 28 were similar to S. cerevisiae elements shown in Figure 2 and were enriched in the S. cerevisiae genes. An additional six matrices were redundantly identified in nearly identical gene groups (namely, Fhl1p targets and ribosomal protein genes) from the same species, and two elements were very similar and identified in the same gene group from *As. nidulans* and*M. grisea*. Thus, in all, 19 novel elements were identified. The complete list of matrices is available in Dataset S47.

#### Positional distribution and spacing of *cis*-sequences

Genes that contained sequences that matched the*S. cerevisiae* position-weight matrices were identified as described above. We then calculated the frequency of each sequence in 50-bp windows upstream of the potential target genes and compared it to the frequency of that element in the corresponding upstream window for all of the genes in that genome. To identify distributions that were statistically different from the background, we identified 50-bp windows that contained a disproportionate number of the *cis*-sequences in the target upstream regions compared to the background, using the hypergeometric distribution presented above, where *i* was the total number of elements identified upstream of the genes in each group, *M* was the number of those elements that fell within a given 50-bp window, *l* was the total number of elements upstream of all of the genes in that genome and *N* was the number of those elements that fell within the same 50-bp window. We considered an element's distribution to be significant if there was at least one 50-bp window with *p* ≤ 0.01; only 5%–10% of the elements had distributions that met this criterion in gene groups other than their putative target genes. We calculated the correlation between element positions in S. cerevisiae and each of the other species by taking all possible pairwise combinations of a *cis*-element's positions in a given S. cerevisiae upstream region and in the orthologous region from other species and plotting these values for each group of coregulated genes (example scatter plots shown in Figures S4 and S5).

Genes that contained sequences that matched the S. cerevisiae Cbf1p and Met31/32p position-weight matrices were identified in each species as described above. The average spacing between Cbf1p and Met31/32p binding sites within the 500 bp-upstream regions of the methionine biosynthesis genes and of all of the genes in each genome was measured by calculating the distance between all pair-wise combinations of the two motifs in each upstream region and taking the average spacing for the respective group of genes.

#### Rpn4p matrix comparisons

To compare the upstream sequences identified in proteasome genes from*S. cerevisiae* and *C. albicans,* and to ensure that the identified sequences were not obtained by sampling bias, we performed the following permutation analysis. We ran MEME on the entire set of upstream regions of 26 proteasome genes with orthologs in both species, using the conservative one-per-sequence model. This produced a “meta-matrix” that identified exactly one putative binding site from each gene, leaving us with a set of exactly 52. We calculated the likelihood-ratio statistic, testing the hypothesis that the sequences were drawn from a single multinomial, or from multinomials estimated separately for each species. In order to test the significance of this statistic, we randomly divided the data into two equal-sized groups 10,000 times, recalculated the statistic, and found that matrix positions 2, 3, and 9 had values of*p* < 0.001. The results were similar when the test was performed on all *cis*-sequences that matched the meta-matrix: These sequences were identified in both species using the S. cerevisiae background model (which identified a list of sequences that was nearly identical to that generated when the C. albicans background model was used to identify motifs from each species).

This set of elements was organized by sequence similarity as follows. Each basepair was represented by a four-dimensional binary vector of indicator variables: G = 1,0,0,0; A = 0,1,0,0; C = 0,0,1,0; T = 0,0,0,1. Each basepair in each 10-mer sequence was replaced by the corresponding vector of indicator variables, translating the 10-mer sequence into a 40-dimensional binary vector. The sequences were organized by hierarchically clustering the binary vectors that represented them, using the program Cluster (Eisen et al. 1998). The organized sequences were visualized using the program TreeView ( available at http://rana.lbl.gov) as shown in Figure S6.

#### Cloning and culture growth

The S. cerevisiae *RPN4* ORF and its orthologs in C. albicans *(orf6.4920)* and *N. crassa (NCU01640.1)* were cloned by PCR from genomic DNA (S. cerevisiae strain S288C, C. albicans strain NIH 3147 [#10231D; American Type Culture Collection, Manassas, Virginia, United States], and N. crassa Mauriceville strain) using Bio-X-act DNA polymerase (BioLine, Boston, Massachusetts, United States). Primers that exactly spanned each ORF (excluding the first ATG) and introduced XmaI and NcoI sites at the 5′ and 3′ ends, respectively, of each PCR product, were used to amplify each ORF. The digested products were cloned into pCAL-n (Stratagene, La Jolla, California, United States) to add an amino-terminal calmodulin-binding protein tag to each protein. In addition, a hybrid protein was generated from the amino-terminal portion of *Sc_ RPN4* (corresponding to nucleotide position 4–1,247) and the DNA binding-domain from C. albicans *orf6.4920* (position 1,235–1,611), guided by Clustal W (Thompson et al. 1994; Chenna et al. 2003) alignments of the proteins. The *orf6.4920* fragment was amplified by PCR, generating an EcoRI site in the amino end of the fragment. The digested fragment was ligated to a natural EcoRI site in*Sc_RPN4* (present in a region of high sequence conservation between the proteins), and the hybrid was cloned into pCAL-n as described above. The wild-type amino acid sequences of Sc_Rpn4p, Ca_Rpn4p, and the hybrid clones were verified by DNA sequencing. (The Mauriceville Rpn4p ortholog had five amino acid differences compared to the published sequence from strain 74A. Because we recovered the identical sequence from multiple independent PCRs, we take this to be the wild-type Nc_Rpn4p for this strain.) Each plasmid was used to transform BL21DE3-RIL E. coli cells (Stratagene).

Yeast overexpression plasmids were constructed by PCR amplification of *Sc_RPN4, Ca_RPN4,* or *Hybrid_RPN* from the above plasmids and cloned into the GAL-inducible expression plasmid pRS-TAP (provided by D. Nix) by homologous recombination and gapped plasmid repair. Reporter constructs were generated by cloning 40-bp fragments that contained either one or five copies of Sequence A or Sequence B upstream of the *HIS3* minimal promoter in pDC204 (provided by D. Y. C.). Yeast strain BY4741 (MAT**a** *his3Δ1 leu2Δ0 met15Δ0 ura3Δ0,* provided by M. Kobor) was transformed with each overexpression construct and each reporter construct. Liquid cultures were grown to mid-log phase and washed three times with synthetic-dropout medium lacking histidine and glucose. Serial culture dilutions were spotted onto solid SC medium lacking uracil, leucine, and histidine, with 2% galactose, and containing 0–15 mM 3-amino triazole (Sigma, St. Louis, Missouri, United States). Photos were taken after growth for 3 d at 30 °C.

#### Protein purification and Biacore measurements

The proteins were purified from bacteria by affinity purification. 250 ml of LB medium containing 50 ng/ml carbenicillin (Sigma) was inoculated with 8 ml of saturated cultures and grown at 37 °C to OD_600_ of approximately 1.0. The cells were induced with 0.3 mM IPTG (Sigma) at 30 °C for 1 h, collected by centrifugation at 4 °C, and flash-frozen in liquid nitrogen. The cells were resuspended in ice-cold 8V calcium binding buffer (50 mM Tris-Cl [pH 7.5], 150 mM NaCl, 1 mM magnesium acetate, 1 mM imidazole, 2 mM calcium chloride, and 1 mM PMSF) and lysed on ice by sonication. The lysate was cleared by centrifugation, and the soluble extract was loaded onto 0.5 ml of calmodulin resin (Stratagene) in a 2-ml column (BioRad, Hercules, California, United States) at 4 °C. The column was washed with 8V calcium binding buffer followed by 8V binding buffer adjusted to 0.5 M NaCl. The resin was eluted with elution buffer (50 mM Tris-Cl [pH 7.5], 0.5 M NaCl, and 2 mM EGTA), and the eluates were flash-frozen and stored at –80 °C.

The interaction of each purified protein with three predicted Rpn4p binding sites was measured using a Biacore 3000 system (Biacore, Piscataway, New Jersey, United States). Complementary 40-nucleotide oligonucleotides were designed, with one oligonucleotide containing a 5′ biotinylated group (Qiagen, Valencia, California, United States). Each of the three sequences contained a different 10-bp core flanked by the same 15 bp that flanked a natural Rpn4p site from the C. albicans orf6.8078 gene: Sequence A ( GCGTGCCAGATAATCGGTGGCAAAACGGAAGAAAAAGTGA); Sequence B ( GCGTGCCAGATAATCGAAGGCAAAACGGAAGAAAAAGTGA); and Sequence C ( GCGTGCCAGATAATCAGTGGCAACACGGAAGAAAAAGTGA). (The flanking sequence did not noticeably contribute to the binding properties, as a 40-bp fragment consisting of the natural Rpn4p site and flanking sequence from the S. cerevisiae gene *PUP2* performed nearly indistinguishably from Sequence A in competition experiments [unpublished data].) The HPLC-purified oligonucleotides were combined at a ratio of 2:1 unbiotinylated:biotinylated oligonucleotides in 10 mM Tris-Cl (pH 7.4), 1 mM EDTA, and 50 mM NaCl, heated to 95 °C for 10 min, and incubated at room temperature overnight. Each double-stranded, biotinylated sequence was bound to one flow cell of an SA sensor chip (Biacore) in HBS buffer (10 mM HEPES [pH 7.4], 150 mM NaCl, 3 mM EDTA, and 0.005% P20) at a flow rate of 10 μl/min. Each cell was coated with roughly the same DNA (approximately 46–56 response units) according to the manufacturer's instructions. The fourth flow cell was not coated with DNA and served as a control. A single cell on a second SA chip was coated in the same way with double-stranded, biotinylated Sequence I ( ACTTGTTCCCGCTCGCTGGAGCTCCTCCAACGACACGGGC), representing an instance of the GGAGCT site and flanking sequence from the N. crassa proteasome gene *NCU06712.1*.

Protein was diluted to 10–100 nM in ice-cold HBS buffer and maintained on ice until injection into the Biacore system. Proteins were passed through four flow cells at a flow rate of 10 μl/min for 90 s at room temperature, then HBS buffer was flowed over the chip at 10 μl/min for 180 s. The protein was desorbed by flowing 0.5% SDS over the chip for 30 s followed by HBS. The kinetics of binding were examined using the Biacore software, and the fit of each calculation was acceptable according to the manufacturer's instructions.

Double-stranded competitor DNA was generated by mixing equimolar amounts of complementary 30-nucleotide fragments, heating to 95 °C for 10 min, and allowing the mixture to cool to room temperature overnight. The DNAs were quantified before and after annealing by replicate absorbance measurements. Four different competitor fragments were used: Sequence D ( CCAGATAATCGGTGGCAAAACGGAAGAAAA), Sequence E ( CCAGATAATCAGTGGCAAAACGGAAGAAAA), and Sequence F ( CCAGATAATCGGTGGCAACACGGAAGAAAA); the fourth sequence, Sequence G, ( CCAGATAATCCTGCATTTGGCGGAAGAAAA) was chosen as the worst-scoring sequence to the Sc_Rpn4p position-weight matrix and served as a negative control. Each fragment was mixed with 50 nM protein at a 1:1 or 5:1 molar ratio (or buffer was added for mock experiments), incubated for 50 min on ice, then injected into the Biacore system, as described above. The maximum response units of each protein binding to the three sequences on the chip were measured using the Biacore software.

## Supporting Information

Dataset S1 List of S. cerevisiae Consensus Transcription Factor Binding Sites (2 KB TXT).

Dataset S2 List of S. cerevisiae Genes in the 264 Gene Groups Identified (339 KB XLS).

Dataset S3 Transcription Factor/Motif vs. Gene Group Relationships Used to Score Enrichment in Figure 2
(22 KB XLS).

Dataset S4 S. cerevisiae Matrices Identified by MEME That Matched Known S. cerevisiae Binding Sites (47 KB XLS).

Dataset S5 *S. cerevisiae–S. castellii* Orthologs (90 KB TXT).

Dataset S6 *S. cerevisiae–S. kluyveri* Orthologs (64 KB TXT).

Dataset S7 *S. cerevisiae–K. waltii* Orthologs (69 KB TXT).

Dataset S8 *S. cerevisiae–A. gossypii* Orthologs (53 KB TXT).

Dataset S9 *S. cerevisiae–C. albicans* Orthologs (74 KB TXT).

Dataset S10 *S. cerevisiae–N. crassa* Orthologs (70 KB TXT).

Dataset S11 *S. cerevisiae–M. grisea* Orthologs (63 KB TXT).

Dataset S12 *S. cerevisiae–As. nidulans* Orthologs (64 KB TXT).

Dataset S13 *S. cerevisiae–Sch. pombe* Orthologs (54 KB TXT).

Dataset S14 Probability of Enrichment of Genes Containing Two or More Copies of S. cerevisiae Consensus Elements within 500 bp Upstream of S. paradoxus Genes (433 KB XLS).

Dataset S15 Probability of Enrichment of Genes Containing One or More Copies of S. cerevisiae Consensus Elements within 500 bp Upstream of S. paradoxus Genes (433 KB XLS).

Dataset S16 Probability of Enrichment of Genes Containing Two or More Copies of S. cerevisiae Consensus Elements within 500 bp Upstream of S. mikatae Genes (433 KB XLS).

Dataset S17 Probability of Enrichment of Genes Containing One or More Copies of S. cerevisiae Consensus Elements within 500 bp Upstream of S. mikatae Genes (433 KB XLS).

Dataset S18 Probability of Enrichment of Genes Containing Two or More Copies of S. cerevisiae Consensus Elements within 500 bp Upstream of S. bayanus Genes (433 KB XLS).

Dataset S19 Probability of Enrichment of Genes Containing One or More Copies of S. cerevisiae Consensus Elements within 500 bp Upstream of S. bayanus Genes (433 KB XLS).

Dataset S20 Probability of Enrichment of Genes Containing Two or More copies of S. cerevisiae Consensus Elements within 500 bp Upstream of S. castellii Genes (416 KB XLS).

Dataset S21 Probability of Enrichment of Genes Containing One or More Copies of S. cerevisiae Consensus Elements within 500 bp Upstream of S. castellii Genes (359 KB XLS).

Dataset S22 Probability of Enrichment of Genes Containing Two or More Copies of S. cerevisiae Consensus Elements within 500 bp Upstream of S. kluyveri Genes (411 KB XLS).

Dataset S23 Probability of Enrichment of Genes Containing One or More Copies of S. cerevisiae Consensus Elements within 500 bp Upstream of S. kluyveri Genes (414 KB XLS).

Dataset S24 Probability of Enrichment of Genes Containing One or More Copies of S. cerevisiae Consensus Elements within 1,000 bp Upstream of K. waltii Genes (411 KB XLS).

Dataset S25 Probability of Enrichment of Genes Containing Two or More Copies of S. cerevisiae Consensus Elements within 500 bp Upstream of K. waltii Genes (411 KB XLS).

Dataset S26 Probability of Enrichment of Genes Containing One or More Copies of S. cerevisiae Consensus Elements within 500 bp Upstream of K. waltii Genes (416 KB XLS).

Dataset S27 Probability of Enrichment of Genes Containing One or More Copies of S. cerevisiae Consensus Elements within 1,000 bp Upstream of A. gossypii Genes (398 KB XLS).

Dataset S28 Probability of Enrichment of Genes Containing Two or More Copies of S. cerevisiae Consensus Elements within 500 bp Upstream of A. gossypii Genes (406 KB XLS).

Dataset S29 Probability of Enrichment of Genes Containing One or More Copies of S. cerevisiae Consensus Elements within 500 bp Upstream of A. gossypii Genes (405 KB XLS).

Dataset S30 Probability of Enrichment of Genes Containing One or More Copies of S. cerevisiae Consensus Elements within 1,000 bp Upstream of C. albicans Genes (404 KB XLS).

Dataset S31 Probability of Enrichment of Genes Containing Two or More Copies of S. cerevisiae Consensus Elements within 500 bp Upstream of C. albicans Genes (404 KB XLS).

Dataset S32 Probability of Enrichment of Genes Containing One or More Copies of S. cerevisiae Consensus Elements within 500 bp Upstream of C. albicans Genes (389 KB XLS).

Dataset S33 Probability of Enrichment of Genes Containing One or More Copies of S. cerevisiae Consensus Elements within 2,000 bp Upstream of N. crassa Genes (399 KB XLS).

Dataset S34 Probability of Enrichment of Genes Containing Two or More Copies of S. cerevisiae Consensus Elements within 1,000 bp Upstream of N. crassa Genes (410 KB XLS).

Dataset S35 Probability of Enrichment of Genes Containing One or More Copies of S. cerevisiae Consensus Elements within 1,000 bp Upstream of N. crassa Genes (383 KB XLS).

Dataset S36 Probability of Enrichment of Genes Containing One or More Copies of S. cerevisiae Consensus Elements within 500 bp Upstream of N. crassa Genes (384 KB XLS).

Dataset S37 Probability of Enrichment of Genes Containing One or More Copies of S. cerevisiae Consensus Elements within 1,000 bp Upstream of M. grisea Genes (381 KB XLS).

Dataset S38 Probability of Enrichment of Genes Containing Two or More Copies of S. cerevisiae Consensus Elements within 500 bp Upstream of M. grisea Genes (405 KB XLS).

Dataset S39 Probability of Enrichment of Genes Containing One or More Copies of S. cerevisiae Consensus Elements within 500 bp Upstream of M. grisea Genes (378 KB XLS).

Dataset S40 Probability of Enrichment of Genes Containing One or More Copies of S. cerevisiae Consensus Elements within 1,000 bp Upstream of *As. nidulans* Genes (403 KB XLS).

Dataset S41 Probability of Enrichment of Genes Containing Two or More Copies of S. cerevisiae Consensus Elements within 500 bp Upstream of *As. nidulans* Genes (408 KB XLS).

Dataset S42 Probability of Enrichment of Genes Containing One or More Copies of S. cerevisiae Consensus Elements within 500 bp Upstream of *As. nidulans* Genes (402 KB XLS).

Dataset S43 Probability of Enrichment of Genes Containing One or More Copies of S. cerevisiae Consensus Elements within 2,000 bp Upstream of *Sch. pombe* Genes (382 KB XLS).

Dataset S44 Probability of Enrichment of Genes Containing Two or More Copies of S. cerevisiae Consensus Elements within 1,000 bp Upstream of *Sch. pombe* Genes (399 KB XLS).

Dataset S45 Probability of Enrichment of Genes Containing One or More Copies of S. cerevisiae Consensus Elements within 1,000 bp Upstream of *Sch. pombe* Genes (372 KB XLS).

Dataset S46 Probability of Enrichment of Genes Containing One or More Copies of S. cerevisiae Consensus Elements within 500 bp Upstream of *Sch. pombe* Genes (379 KB XLS).

Dataset S47 Significant MEME Matrices Trained on 500-bp or 1,000-bp Upstream Regions of Genes from Non-S. cerevisiae Species (42 KB TXT).

Dataset S48 The *p*-Values of Enrichment Measured for Species-Specific MEME Matrices (11 KB TXT).

Dataset S49 The Number of Orthologs Identified in Each Species in Each Gene Group (19 KB XLS).

Figure S1 The Enrichment Measured for Randomized Consensus Sequences in Target Gene Group Is Not Statistically Significant Consensus sequences identified by enrichment in Figure 2 were randomized, and the enrichment of the randomized sequence in the denoted gene group was scored. An orange box indicates that the corresponding gene group was enriched for genes containing the randomized sequence, according to the key at the bottom of the figure. Notably, none of the randomized sequences was enriched with *p* < 2 × 10^–4^ in the denoted gene group from any species.
(1.1 MB TIF).

Figure S2 Significant Enrichment Measured for Randomized Upstream Sequences in Random Gene Groups Is Not Consistent across Species Fifteen of the randomized sequences shown in Figure S1 were enriched below the cutoff of *p* < 2 × 10^–4^ in any gene group. However, the enrichment was not consistent across species. Only two randomized sequences were enriched in the same gene group from two species, although the enrichment pattern did not correlate with the species tree. Thus, randomized sequences are enriched with different characteristics than the functional consensus sequences shown in Figure 2
(898 KB TIF).

Figure S3 The Enrichment Measured for S. cerevisiae Consensus Sequences Is Tolerant of Noise in Each Gene Group Our ability to detect conserved *cis*-regulatory elements in other species requires identification of orthologs of the coregulated S. cerevisiae genes. We wondered how our enrichment-based method would be affected if incorrect orthologs were assigned to individual S. cerevisiae genes, thereby producing “noise” in the gene groups. To test the sensitivity of our method to this type of noise, we performed the following gene replacement control: For each group of S. cerevisiae genes, we performed 100 trials in which 0%–100% of the genes in each group were randomly selected and replaced with random S. cerevisiae genes. The number of trials in which the *p* of enrichment was below our cutoff of *p* < 2 × 10^–4^ was scored with an orange box, according to the key shown at the bottom of the figure. Nearly all of the *cis*-elements could be identified in their respective gene groups despite some amount of “noise” in the gene group.
(864 KB TIF).

Figure S4 Correlation between Rpn4 Element Positions in S. cerevisiae Upstream Regions and Orthologous Regions from Other Species Positions of Rpn4p elements upstream of each S. cerevisiae proteasome gene (x axis) were plotted against the positions of Rpn4p elements upstream of the orthologous proteasome gene from each of the other species (y axis). The linear fit is shown in the upper right corner of each plot.
(685 KB TIF).

Figure S5 Correlation between MCB Element Positions in S. cerevisiae Upstream Regions and Orthologous Regions from Other Species Positions of MCB elements upstream of S. cerevisiae G1-phase genes (x axis) were plotted against the positions of MCB elements upstream of the orthologous G1-phase gene from each of the other species (y axis). The linear fit is shown in the upper right corner of each plot.
(767 KB TIF).

Figure S6 Position-Weight Matrices and C*is*-Sequences Found Upstream of Proteasome Genes Sequences within 500 bp upstream of the S. cerevisiae or C. albicans proteasome genes that matched the species-independent meta-matrix were identified as described.
(A) The identified sequences were used to generate sequence logos (Crooks et al. 2004) to represent the set of *cis*-sequences from S. cerevisiae (top) or from C. albicans (bottom). The height of each letter represents the frequency of that base in that position of the matrix. Positions in the matrices that are statistically different (see Materials and Methods for details) are indicated with an asterisk.
(B) Examples of the species-independent meta-matrix found upstream of S. cerevisiae proteasome genes (shown in red) and C. albicans proteasome genes (shown in blue) were pooled and organized by a hierarchical clustering method, as described in Materials and Methods. The sequences found upstream of S. cerevisiae genes only (red bar), C. albicans genes only (blue bar), or both the S. cerevisiae and C. albicans proteasome genes (black bar) are indicated, along with the consensus sequence representing each denoted group.
(1.1 KB TIF).

Figure S7 3-Amino-Triazole Resistance Due to Sc_Rpn4p Activity S. cerevisiae cells harboring a *HIS3* reporter gene with either a minimal promoter (left), minimal promoter + Sequence A (middle), or minimal promoter + Sequence B (right), and overexpressing Sc_Rpn4p from a galactose-inducible promoter, were grown on 0 mM, 1 mM, 5 mM, or 15 mM His3p inhibitor 3-amino-triazole. Two serial dilutions of each strain were plated for each drug concentration. The level of drug resistance is indicative of the level of *HIS3* expression (Guthrie and Fink 2002).
(631 KB TIF).

## Abbreviation

MCB: Mlu1 cell-cycle box
ORF: open reading frame
