# Supplementary figures and images for: Conservation and Evolution of Cis-Regulatory Systems in Ascomycete Fungi
Source: PLoS Biol. 2004 Nov 9;2(12):e398. doi: 10.1371/journal.pbio.0020398 (PMC526180; doi:10.1371/journal.pbio.0020398)

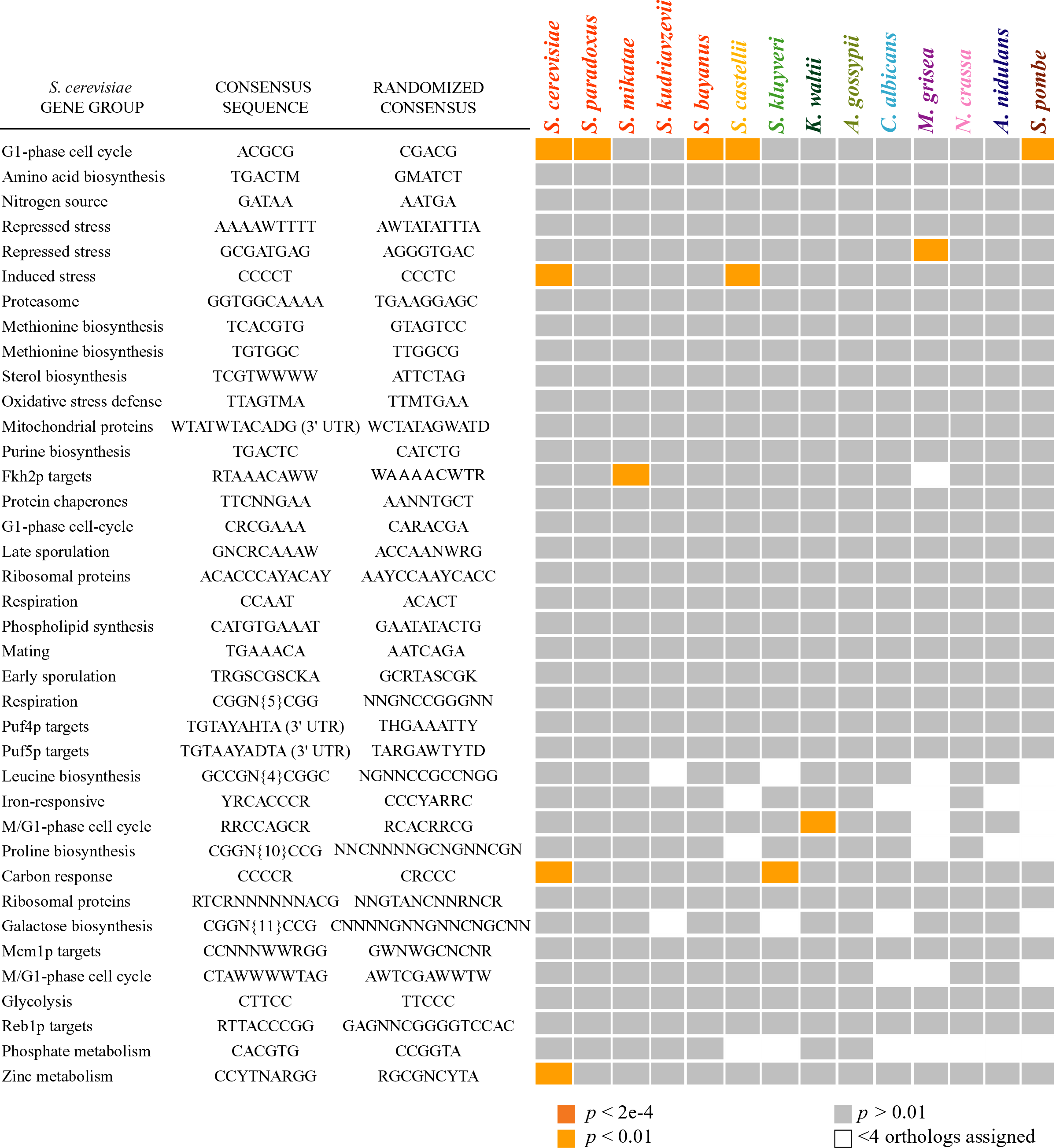

Supplement: Figure S1 — Consensus sequences identified by enrichment in Figure 2 were randomized, and the enrichment of the randomized sequence in the denoted gene group was scored. An orange box indicates that the corresponding gene group was enriched for genes containing the randomized sequence, according to the key at the bottom of the figure. Notably, none of the randomized sequences was enriched with p < 2 × 10^–4^ in the denoted gene group from any species. (1.1 MB TIF). [file pbio.0020398.sg001.tif]

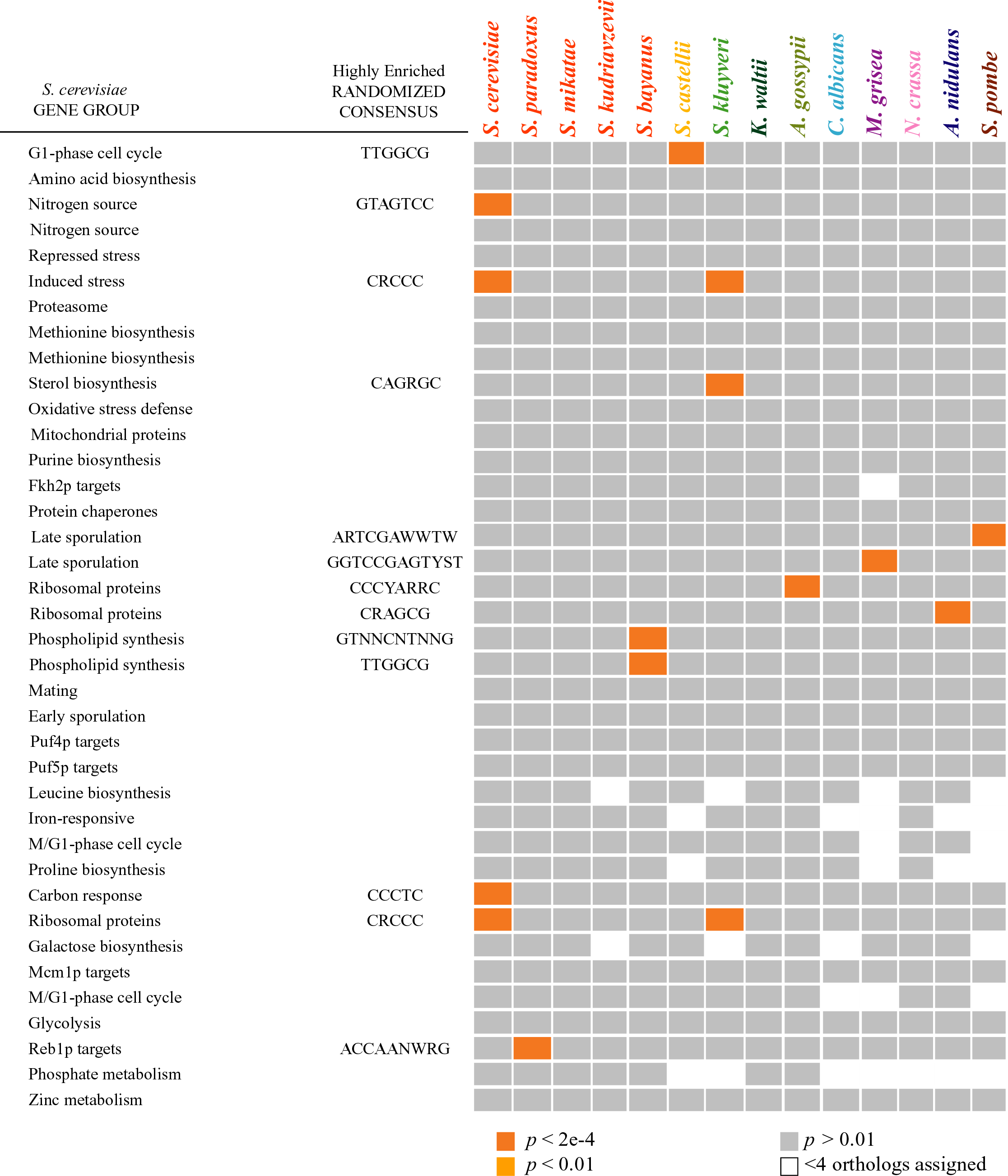

Supplement: Figure S2 — Fifteen of the randomized sequences shown in Figure S1 were enriched below the cutoff of p < 2 × 10^–4^ in any gene group. However, the enrichment was not consistent across species. Only two randomized sequences were enriched in the same gene group from two species, although the enrichment pattern did not correlate with the species tree. Thus, randomized sequences are enriched with different characteristics than the functional consensus sequences shown in Figure 2 (898 KB TIF). [file pbio.0020398.sg002.tif]

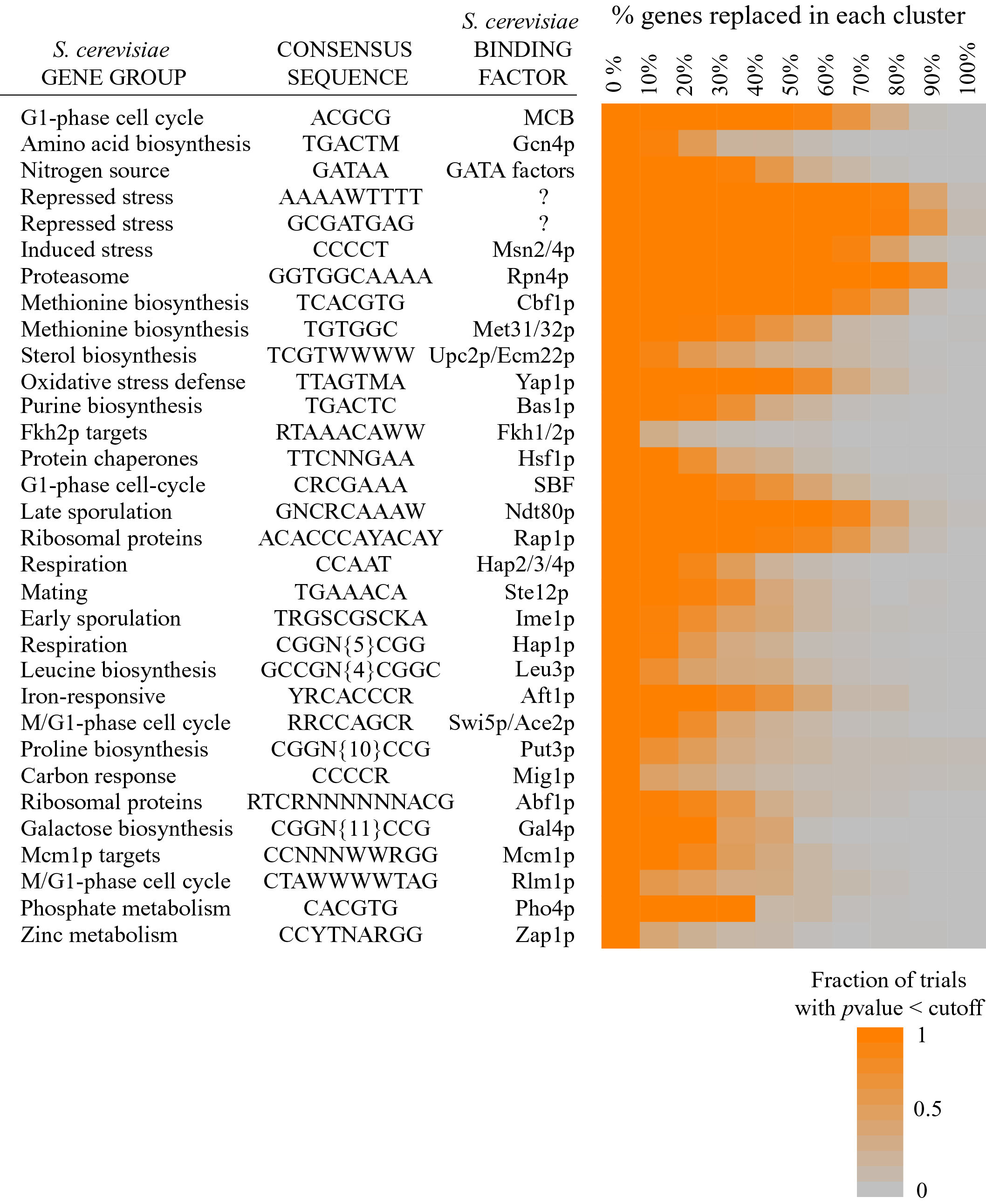

Supplement: Figure S3 — Our ability to detect conserved cis-regulatory elements in other species requires identification of orthologs of the coregulated S. cerevisiae genes. We wondered how our enrichment-based method would be affected if incorrect orthologs were assigned to individual S. cerevisiae genes, thereby producing “noise” in the gene groups. To test the sensitivity of our method to this type of noise, we performed the following gene replacement control: For each group of S. cerevisiae genes, we performed 100 trials in which 0%–100% of the genes in each group were randomly selected and replaced with random S. cerevisiae genes. The number of trials in which the p of enrichment was below our cutoff of p < 2 × 10^–4^ was scored with an orange box, according to the key shown at the bottom of the figure. Nearly all of the cis-elements could be identified in their respective gene groups despite some amount of “noise” in the gene group. (864 KB TIF). [file pbio.0020398.sg003.tif]

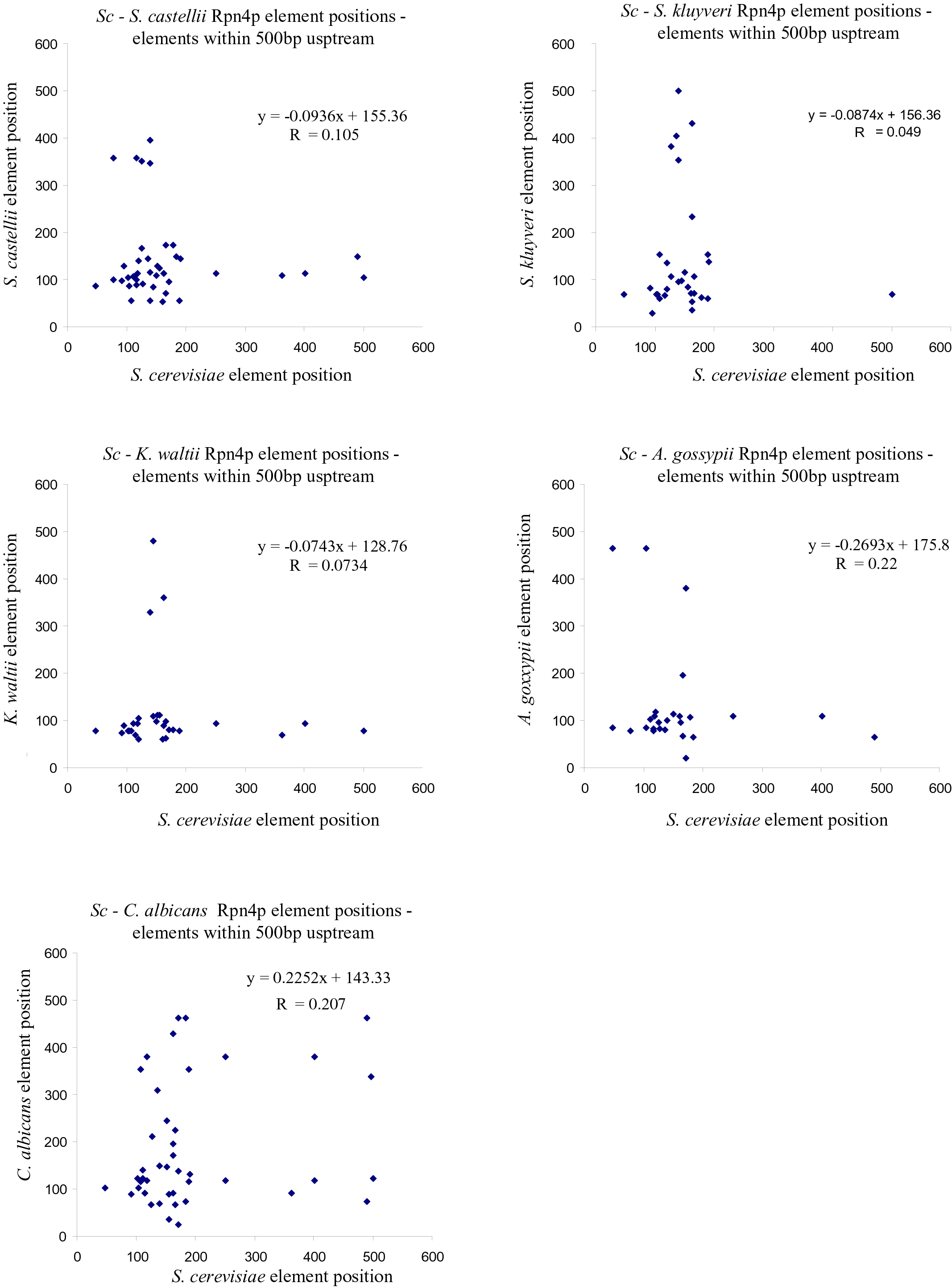

Supplement: Figure S4 — Positions of Rpn4p elements upstream of each S. cerevisiae proteasome gene (x axis) were plotted against the positions of Rpn4p elements upstream of the orthologous proteasome gene from each of the other species (y axis). The linear fit is shown in the upper right corner of each plot. (685 KB TIF). [file pbio.0020398.sg004.tif]

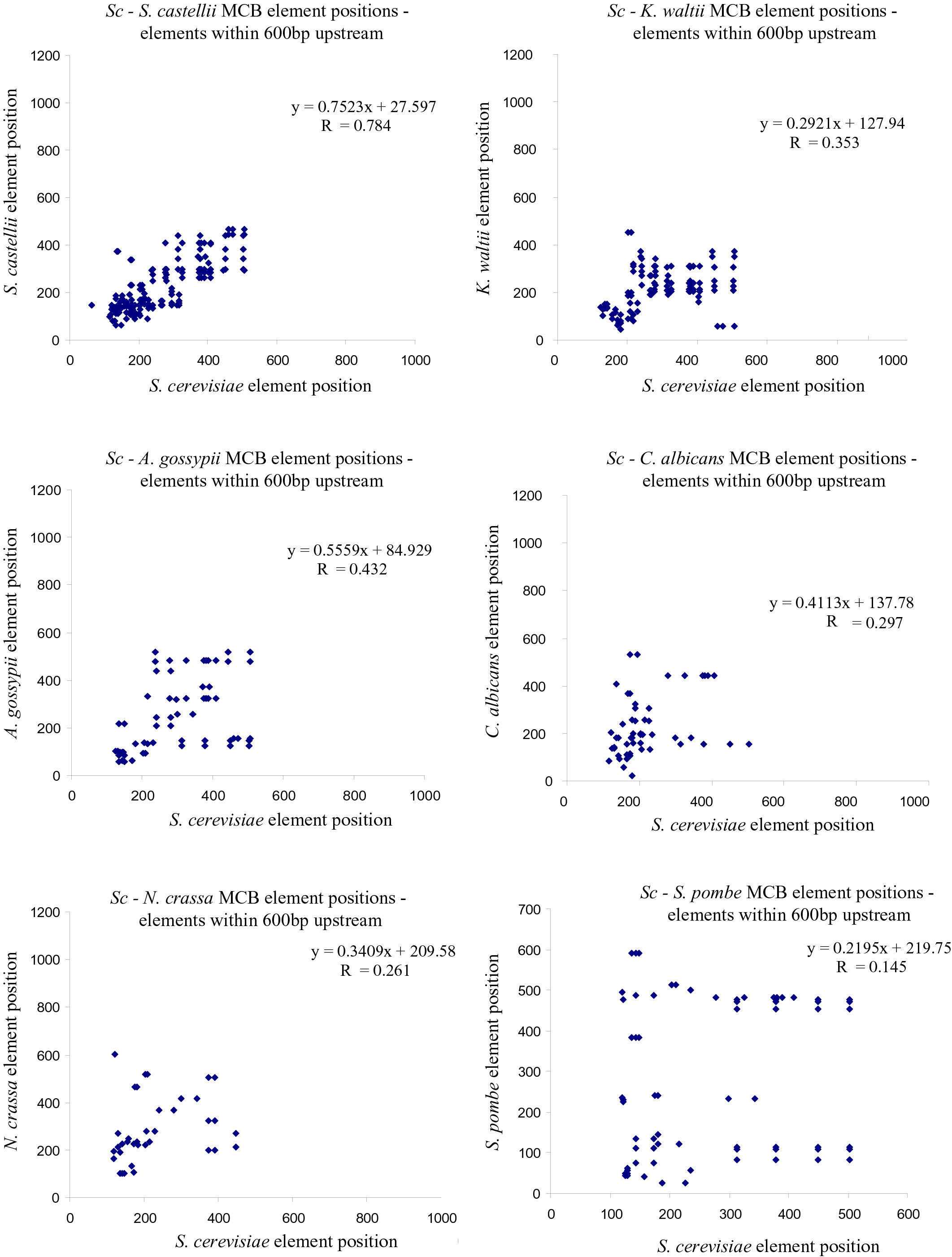

Supplement: Figure S5 — Positions of MCB elements upstream of S. cerevisiae G1-phase genes (x axis) were plotted against the positions of MCB elements upstream of the orthologous G1-phase gene from each of the other species (y axis). The linear fit is shown in the upper right corner of each plot. (767 KB TIF). [file pbio.0020398.sg005.tif]

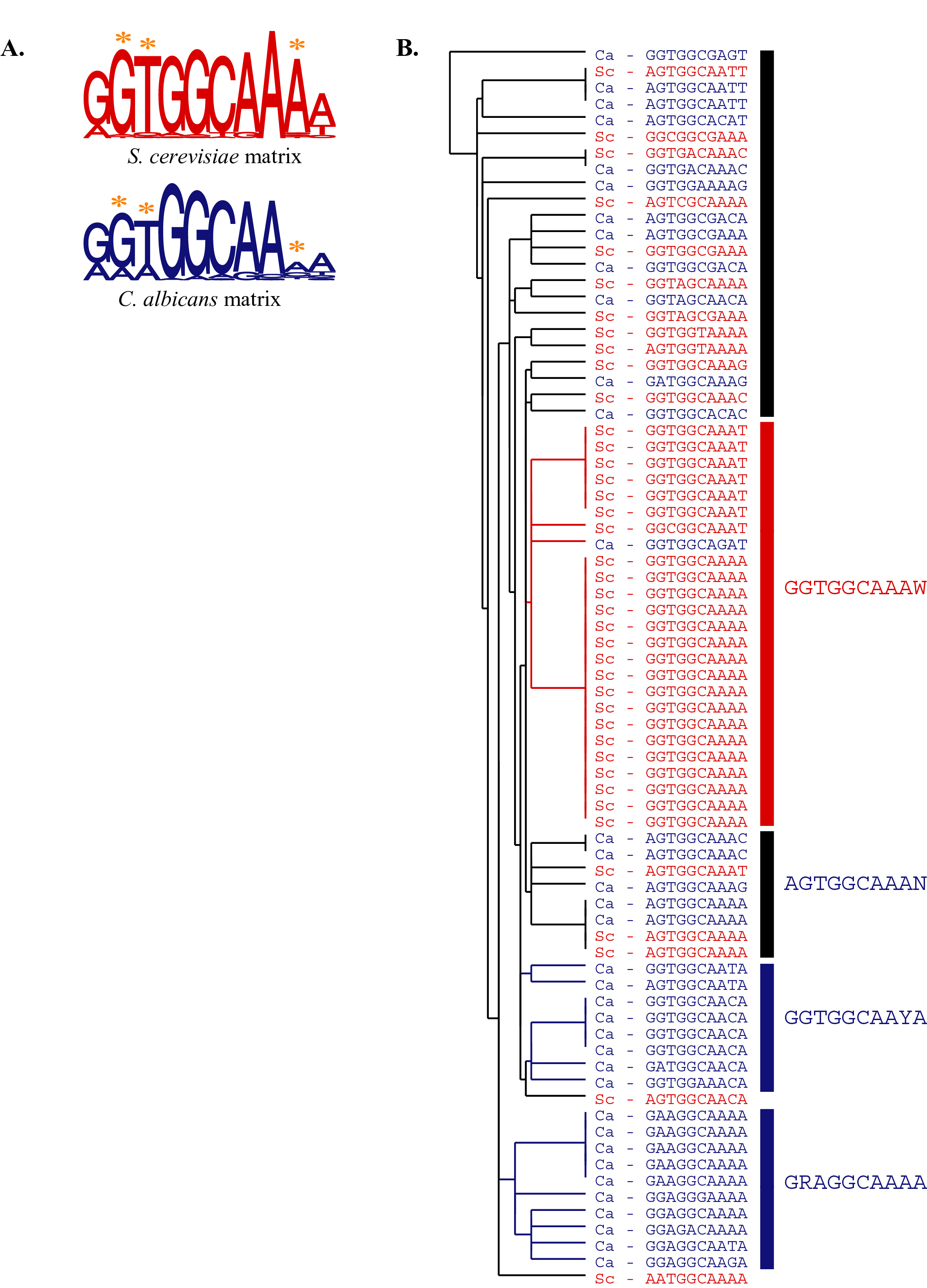

Supplement: Figure S6 — Sequences within 500 bp upstream of the S. cerevisiae or C. albicans proteasome genes that matched the species-independent meta-matrix were identified as described. (A) The identified sequences were used to generate sequence logos (Crooks et al. 2004) to represent the set of cis-sequences from S. cerevisiae (top) or from C. albicans (bottom). The height of each letter represents the frequency of that base in that position of the matrix. Positions in the matrices that are statistically different (see Materials and Methods for details) are indicated with an asterisk. (B) Examples of the species-independent meta-matrix found upstream of S. cerevisiae proteasome genes (shown in red) and C. albicans proteasome genes (shown in blue) were pooled and organized by a hierarchical clustering method, as described in Materials and Methods. The sequences found upstream of S. cerevisiae genes only (red bar), C. albicans genes only (blue bar), or both the S. cerevisiae and C. albicans proteasome genes (black bar) are indicated, along with the consensus sequence representing each denoted group. (1.1 KB TIF). [file pbio.0020398.sg006.tif]

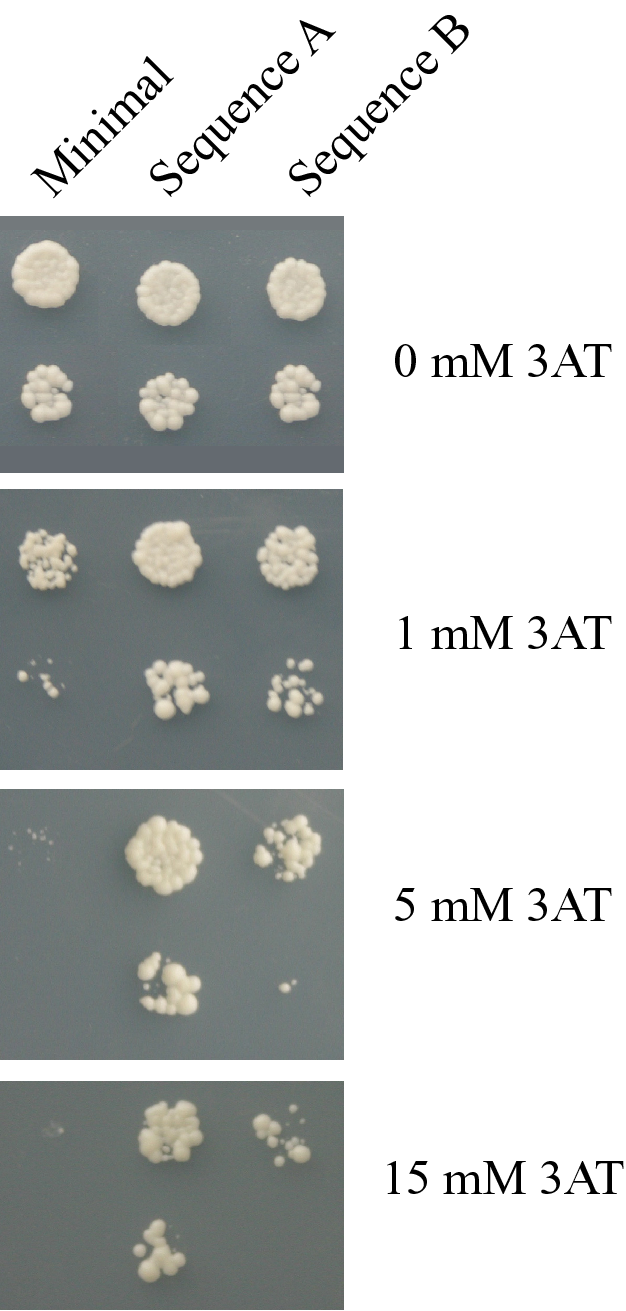

Supplement: Figure S7 — S. cerevisiae cells harboring a HIS3 reporter gene with either a minimal promoter (left), minimal promoter + Sequence A (middle), or minimal promoter + Sequence B (right), and overexpressing Sc_Rpn4p from a galactose-inducible promoter, were grown on 0 mM, 1 mM, 5 mM, or 15 mM His3p inhibitor 3-amino-triazole. Two serial dilutions of each strain were plated for each drug concentration. The level of drug resistance is indicative of the level of HIS3 expression (Guthrie and Fink 2002). (631 KB TIF). [file pbio.0020398.sg007.tif]
